# An Information Theoretic Clustering Approach for Unveiling Authorship Affinities in Shakespearean Era Plays and Poems

**DOI:** 10.1371/journal.pone.0111445

**Published:** 2014-10-27

**Authors:** Ahmed Shamsul Arefin, Renato Vimieiro, Carlos Riveros, Hugh Craig, Pablo Moscato

**Affiliations:** 1 Centre for Bioinformatics, Biomarker Discovery and Information-Based Medicine, The University of Newcastle, Callaghan, New South Wales, Australia; 2 Centre for Literary and Linguistic Computing, The University of Newcastle, Callaghan, New South Wales, Australia; Massachusetts Institute of Technology, United States of America

## Abstract

In this paper we analyse the word frequency profiles of a set of works from the Shakespearean era to uncover patterns of relationship between them, highlighting the connections within authorial canons. We used a text corpus comprising 256 plays and poems from the 16th and 17th centuries, with 17 works of uncertain authorship. Our clustering approach is based on the Jensen-Shannon divergence and a graph partitioning algorithm, and our results show that authors' characteristic styles are very powerful factors in explaining the variation of word use, frequently transcending cross-cutting factors like the differences between tragedy and comedy, early and late works, and plays and poems. Our method also provides an empirical guide to the authorship of plays and poems where this is unknown or disputed.

## Introduction

Authors develop and eventually evolve a highly individual literary style throughout their productive life [1]. One widely known form that this language individuation takes is systematic alteration in the relative frequencies of particular words, phrases or tokens. Such variation can provide a strong basis for classification of authorship. The idea that this sort of variation occurs even in the use of the most common words, and that frequencies of these words could serve for authorship attribution, dates back to the 1960s, specifically, the statistical work of Ellegard on a set of anonymous eighteenth-century published letters [2] and of Mosteller and Wallace on the jointly authored Federalist papers [3], but was developed to a regular technique by Burrows in the 1980s. Burrows pioneered the use of multivariate techniques like Principal Component Analysis on sets of frequencies of very common words to attribute disputed texts [4], [5], and similar methodologies have since been widely used [6]–[8].

Researchers have also explored the usefulness for attribution of slightly less common words, which tend to be lexical words rather than function words, and of very rare words [9]–[11]. Authorship studies using quantitative methods, most often relying on word frequencies, but also exploiting letter and word-grams, and punctuation, are now well established. The field, referred to as stylometry and computational stylistics, has been the object of study of several works [12]–[14], being, perhaps, one of the most important topics within digital humanities scholarship.

It is also worth noting that in many operations with natural language (such as topic detection and information retrieval), the usual practice is to discard the most common words (so-called *stop words* [15], [16]). In quantitative authorship attribution the usual practice is to select a group of word-probabilities for analysis, either by overall frequency or by relative probabilities between authors [14]. Researchers have debated the merits of *culling* word lists according to various rules as opposed to using all the words within a given category [9], [17], [18]. In a previous research, we demonstrated that such consideration can potentially reflect the authors' individuality and style [19]. In contrast, this research is carried out considering all the words –including stop words. We present here a partitioning of the complete graph of 256 plays and poems, depicting a taxonomy of the works, where we verify the results by statistically comparing against randomised groupings. The authors' tendency to over-utilise or avoid particular words or phrases containing them effectively guided us to postulate authors for works previously classified as “uncertain”. In some cases we acknowledge that a similarity in topic, rather than authorship, may be the best explanation for a close relationship between works.

The system we present, based on word probabilities, the Information Theoretic measure Jensen-Shannon divergence (JSD), and a graph partitioning clustering algorithm, is unsupervised, in the sense of having no input from authorship, genre or any other metadata, and non-parametric, automatically determining the number and composition of the outcome groups. The relationships summarised in the clustering include all the various known and unknown sources of similarity and dissimilarity between these works. The clustering outcome demonstrated distinctive predominance of authorial affinities in the corpus and the mode of the work (non-dramatic poetry versus play) is also clearly differentiated.

## Materials and Methods

### Data set

In this work, we utilised a text corpus containing 256 plays and poems from the Shakespearean era, containing texts of authorship from the 16th and 17th centuries. The machine-readable texts of the plays and poems are held in an archive in the Centre for Literary and Linguistic Computing at The University of Newcastle. They have been assembled over some years by editing versions available from commercial online collections like Literature Online (Chadwyck-Healey) or from other sources such as keyboarding from early printed versions. There is no comprehensive collection of electronic texts of these works in the public domain. We used a software tool called *Intelligent Archive* (IA) by Craig and Whipp [20] to pre-process the corpus. The IA creates sub-corpora and generates counts of word-forms according to a parameterised user input, taking into account the variations in spelling commonly found in 16th and 17th century plays and poems, in addition to facilitating disambiguation of words by both context and frequency. The tool identified in total a set of approximately 66,907 unique words in the 256 texts. IA calculated the frequency of each of the aforementioned 66,907 words in each work and stored the final outcome in the form of a 66,907×256 matrix (File S1).

### Data Clustering

We utilised an unsupervised graph-based clustering method called MST-kNN to cluster plays and poems in the data set generated by the Intelligent Archive. The non-parametric MST-kNN algorithm [21] (see also its external-memory variant in [22] and a GPU-based data-parallel variant in [23], [24] and several applications in [25]–[28]), takes as input a weighted undirected complete graph (*G*) and computes two proximity graphs: a minimum spanning tree (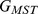) and a k-nearest neighbour graph (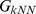), where the value of 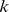 is automatically determined by Equation 1.
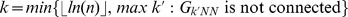
(1)


Subsequently, the algorithm inspects all edges in 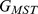. If for a given edge 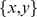 neither 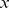 is one of the 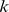 nearest neighbours of 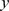, nor 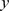 is one of the 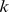 nearest neighbours of 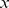, the edge is eliminated from 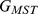. This results in a new graph 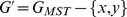. Since 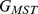 is a tree, after the first edge is deleted, 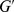 becomes a forest. The algorithm recursively applies the same procedure to each sub-tree in 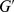 until no further partition is possible; the value of 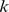 is re-adjusted to 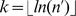 in each iteration, where 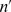 is current number of nodes in the sub-tree. The final partition of the nodes of 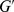 is the result of the clustering algorithm.

We started our analysis by producing a complete weighted graph (distance matrix File S2) where all plays and poems are connected to each other. The weights of the connection between two works, i.e. the edge weights of the graph, corresponded to the pair-wise *Jensen-Shannon divergence* (JSD) between the frequencies of words in these two documents. The JSD is a metric of similarity between distributions, and is defined for two probability distributions 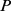 and 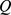 as follows (Equation 2):
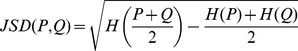
(2)where 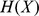 is Shannon's information entropy for distribution 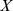, defined by Equation 3.
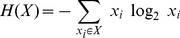
(3)


Here, 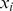 is the probability of occurrence of word 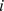 in document 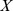, and 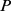 and 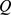 refer, in our case, to the 66,907 word frequencies in two arbitrary documents in the data set. We then applied the MST-kNN algorithm to produce the initial clustering.

In order to identify the core interactions amongst plays and poems in the initial clusters, we identified all the maximal cliques on the kNN graph of works. We then selected the cliques of the largest three sizes (a subgraph hereto named as the *top-3 maximal cliques*); this is the subgraph formed by the union of all the maximal cliques (of size 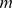) present in the graph, plus all the maximal cliques of size 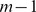 and 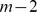. As a clique of size 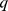 is formed by 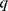 cliques of size 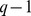, by this procedure we attempted to collect larger structures present in the graph that were lacking a few edges to become a clique of a larger size. These clique-like structures are also known as *paracliques* in the literature [29].

We computed the cliques using the *igraph* package for R [30], applied on the kNN graph computed using the same distance matrix used to find clusters. The number of nearest neighbours was set to 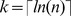, for 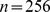 and then we identified cliques of size 8, 7 and 6 on the 6-NN graph. Once the cliques were found, they were projected on the MST-kNN graph in order to identify core interactions in each cluster.

### Statistical Significance

To verify whether our clustering was indeed identifying meaningful association affinities in the corpus, we conducted a random permutation test. To do this, we first assigned scores to tokens (e.g., authors) based on their associations in the clustered graph. Each token was assigned two different scores: *self* and *diff*, where the *self score* represents the number of connections between same token types and *diff score* represents the number of connections between different token types. Scores were halved in the cases of collaborations. Further, in the works that are collaborations of many, we considered the first and all the “others” as individual entities. A detailed example on how we computed these scores on two connected nodes (considering various configurations) is given in Table 1.

**Table 1:** Scoring rule for the tokens in graph node labels.

Graphs/Nodes				A1		A2		A3		A4	
Node1		Node2		self	diff	self	diff	self	diff	self	diff
A1		A1		1	0	0	0	–	–	–	–
A1		A2		0	1	0	1	–	–	–	–
A1	A2	A1		0.5	0.5	0	0.5	–	–	–	–
A1	A2	A3		0	0.5	0	0.5	0	1	–	–
A1	A2	A3	A4	0	0.5	0	0.5	0	0.5	0	0.5

We associated two different attributes, *self* and *diff* with each token e.g., an author, play/poem, genre or their combination. For example, in regards to the authors, when one work was connected with another completely written by him/herself, the *self* score was increased by 1 and the same applied for the *diff* score. However, when the work had shared authorship, we broke down the scoring and allocated halves to each of the attributes. Where one author collaborated with many (e.g., Shakespeare and others) we considered the “other” as a single authorial entity.

**Figure 1:**
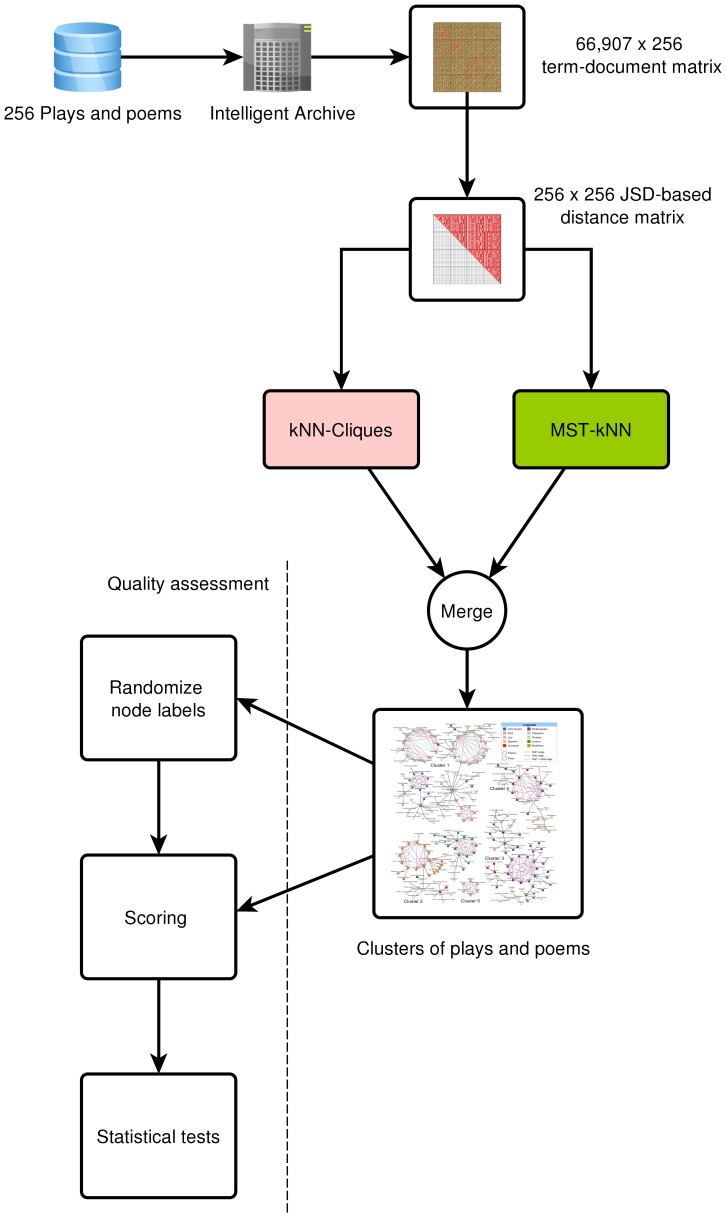
Flowchart of the proposed Information Theoretic method for clustering plays and poems. The software tool Intelligent Archive generated a set of approximately 66,907 unique words from 256 Shakespearean-era plays and poems and computed the frequency of each of the words in each work, in the form of a 66,907×256 matrix. Then the MST-kNN + kNN Clique method generated the clusters using this term-document matrix and an Information theoretic measure, Jensen-Shannon Divergence.

We used two different configurations to assess the significance of our clustering results. First, we used the *Wilcoxon signed-rank test* [31] where we compared the mean rank of differences between the number of edges connecting works of same and different authorship in our observed data and random labelling. In the second configuration we performed the same but using the *Kruskal-Wallis test* [31]. Finally, in our third configuration, we considered each of the differences from the permutations as an independent sample and performed a *Kruskal-Wallis test* on the 1001 samples (observed data and 1000 permutations of the clustering outcome graph). All tests were conducted using the standard *stats* package in R. The complete method for data clustering and assessment is depicted in Figure 1.

To further evaluate the significance of our clustering with respect to the choice of distance, we computed distance matrices using four other popular metrics: Cosine, Pearson's, Spearman's and a robust metric (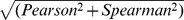). We then re-clustered the 256 works with these metrics, and performed the Wilcoxon and Kruskal-Wallis tests on the randomised permutations, for different choices of tokens and their combinations. The attributes tested where: author, genre, mode (play/poem) and the combinations author + genre, author + mode, genre + mode.

### kNN Classifier

We also performed a “clustering-free” performance benchmark to further investigate if indeed this representation of works (a probability distribution of words) and the use of perhaps the simplest of all classifiers (a 3-nearest neighbour classifier) can perform well at authorship attribution. This classification step did not include any *ad hoc* training phase and was non-parametric. Given a certain work, we looked at assigning authorship based on the authorship of the majority of its 3 neighbours. We counted the number of correctly assigned authors, the number of mistakes, and the number of times in which we did not reach a majority consensus (at least 2 out of 3, labelled as ‘Undecided’).

## Results

**Figure 2:**
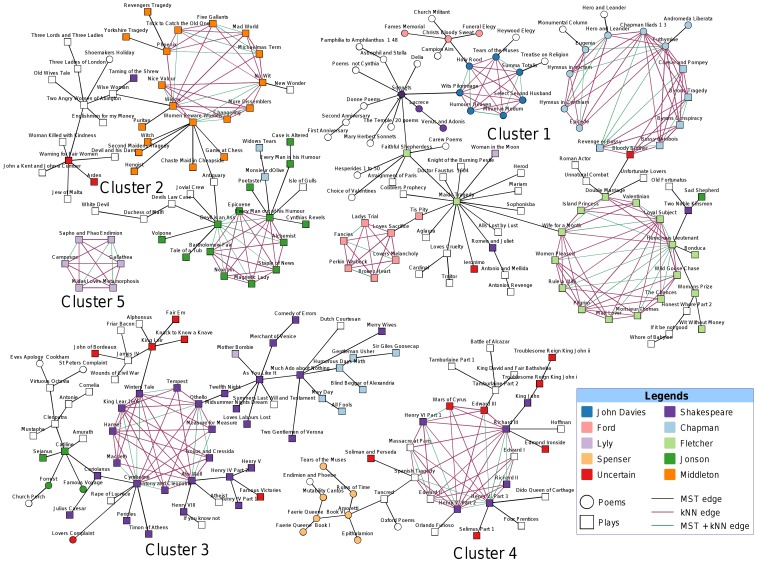
Clustering outcome of the MST-kNN + kNN Clique graph partitioning algorithm on the distance matrix produced by using pair-wise Jensen-Shannon divergence of the works' token frequencies. The top 3 maximal cliques on the kNN graph of works were identified. The number of nearest neighbours was set as 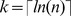, where *n* = 256 and once the cliques were found, they were projected on the MST-kNN outcome in order to identify core interactions in each cluster. A total of eight highly connected networks were formed for the Chapman, Fletcher, Middleton, Jonson, John Davies, Ford, Shakespeare, Lyly and unknown authors.

**Figure 3:**
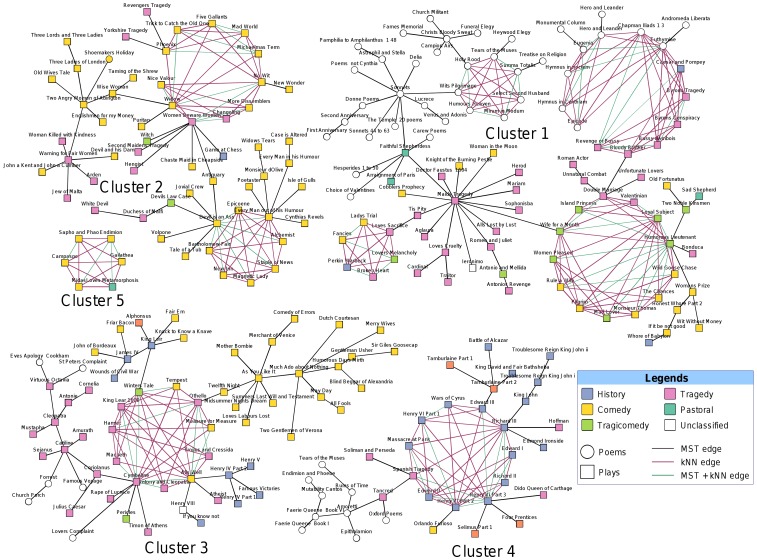
Clustering outcome of the MST-kNN + kNN Clique graph partitioning algorithm on the distance matrix produced using pair-wise Jensen-Shannon divergence, where the colours represent the genres of the works. Association among genres like Comedy, History or Tragedy is clearly visible.

The method divided the 256 plays and poems from 60 authors and a separate category of works of unknown authorship into five cluster components. The outcome is presented in Figure 2, which is coloured by the major contributing authors. A variant of this representation is shown in Figure 3, depicting the genres of the works. Based on similarity to core subgroups and number of connections to neighbouring authors on the identified clusters, we assigned a plausible authorship for the 17 uncertain works in the corpus (Table 2). A discussion of our findings follows.

**Table 2:** Authorship similarity in the Information Theory based clustering.

Uncertain works	Authorship based on the MST-kNN + kNN cliques	Connected/similar to
Bloody Brother	Chapman	Revenge of Bussy, Caesar and Pompey, Byron's Tragedy, Byron's Conspiracy, Bussy d'Ambois (Chapman), Double Marriage (Fletcher and Massinger)
Ieronimo	Beaumont and Fletcher (weak association)	Maids Tragedy (Beaumont and Fletcher)
Warning for Fair Women	Haughton (weak association)	Arden, Two Devil and his Dame (Haughton), Angry Women of Abington (Henry Porter), John a Kent and John a Cumber (Munday), Jew of Malta (Marlowe), Woman Killed with Kindness (Heywood)
Arden	-	Warning for Fair Women
John of Bordeaux	Greene	James IV (Greene)
Fair Em	-	King Leir
King Leir	Greene/Shakespeare (weak association)	Alphonsus (Greene), James IV (Greene), Winters Tale (Shakespeare), Fair Em, Knack to Know a Knave
Knack to Know a Knave	-	King Leir
Lovers Complaint	Shakespeare	Cymbeline (Shakespeare)
Famous Victories	Shakespeare	Henry IV Part 2 (Shakespeare and others)
Soliman and Perseda	Kyd (weak association)	Spanish Tragedy (Kyd)
Selimus Part 1	Shakespeare and others	Henry VI Part 3 (Shakespeare and others)
Wars of Cyrus	Shakespeare and others	Henry VI Parts 1, 2, 3 (Shakespeare and others), Richard III (Shakespeare), Edward III
Edward III	Shakespeare and others	Henry VI Parts 1, 2, 3 (Shakespeare and others), Richard III (Shakespeare), Wars of Cyrus
Edmond Ironside	Shakespeare	Richard III (Shakespeare)
Troublesome Reign King John I	Shakespeare	King John (Shakespeare)
Troublesome Reign King John II	Shakespeare	Troublesome Reign King John I

The authorship is determined by looking at the similarity and number of connections to neighbouring works. A weak association is noted when a work is connected to uncertain or multiple authorship. The attribution is further investigated using the 3NN classification in Table 4.

*Cluster 1* was formed by 96 plays and poems, which accumulated works from a total of 38 authors. The four major contributing authors of this cluster, **Fletcher**, **Chapman**, **Ford** and **John Davies** formed the four networks of nearest neighbours, i.e., the ring-shaped structures in Figure 2. Even though the cluster was formed by heterogeneous contributions from various authors, it can still highlight some interesting facts about the authorships of the comprising plays and poems.

For example, the poem *Funeral Elegy* by Ford was claimed for Shakespeare from the late 1990s onwards, appearing in some collected editions of Shakespeare, but is now accepted as by John Ford [32], and duly appeared in a tree of Ford poems in the current analysis.

Further, in Chapman's network of nearest neighbours, only 13 out of his 21 contributions in the data set appeared, while the networks formed by the plays and poems of Fletcher, Ford and John Davies agglomerated all of their contributions, which suggests a higher level of associations than among these particular works of Chapman. Moreover, only the poems, four of his tragedies and a classical history clustered into this network and all of his comedies remained elsewhere, six as a part of a larger comedy tree in Cluster 3, and two connected to Jonson's nearest neighbour network of works in Cluster 2.

This cluster also unveiled the possibility of attributing a play of uncertain authorship, titled *The Bloody Brother*, as it appeared closely related to some Chapman works and formed a part of his network of nearest neighbours. However, this tragedy is also connected to the Fletcher network; specifically to a play by Fletcher and Massinger. It is the only work not securely attributed to Chapman. It is sometimes known by an alternative title, *Rollo, Duke of Normandy*, and its authorship is much disputed, with Fletcher and Massinger being the most serious candidates [33]; Chapman is mentioned as a possible author but little textual evidence has been offered for this [34]. The strength of the affinities with Chapman in the current analysis suggests that he could be reconsidered as one of the primary authors.

Cluster 2 was formed by 56 plays and poems from a total of 19 authors. The two major contributing authors of this cluster, **Middleton** and **Jonson**, produced the two networks of nearest neighbours. The network formed by Middleton's works agglomerated all of his contributions in the data set, while the Jonson's network was formed by 14 out of his 19 works.

Two works of uncertain authorship (*Warning for Fair Women* and *Arden*) appeared in this cluster, however not with any of the nearest neighbour networks. The play titled *Warning for Fair Women* demonstrated weak similarities with *Jew of Malta* by Marlowe, *Woman Killed with Kindness* by Heywood, *John a Kent and John a Cumber* by Munday, *Devil and his Dame* by Haughton and *Two Angry Women of Abington* by Henry Porter, which was also connected to *Arden*.

It is important to note that appearance of Jonson and Middleton in this same cluster also has a meaningful placement, as they are the two representative of the *city comedy* genre, a satirical approach to describe stories of characters seeking fortune and love in Renaissance London [35].

*Cluster 3*, formed by 63 plays and poems, received contributions from a total of 23 authors. The major contributing author, **Shakespeare**, formed the only network of nearest neighbours, which accumulated most of Shakespeare's individual contributions (23 out of 31 works in the data set). However, some of the works by Chapman (six plays) and Jonson (two plays and two poems) also appeared here and, more importantly, three (*King Leir*, *Lovers Complaint* and *Famous Victories*) out of six works of uncertain authorship in this cluster closely grouped with Shakespeare's works. It may be noted that *King Leir* is one of the sources for Shakespeare's *King Lear* [36], and it is safe to assume that in this case it is an overlap in subject matter, rather than common authorship, that connects this play to the Shakespeare nearest neighbour network.

Furthermore, the anonymous poem titled *Lover's Complaint* was closely attached to the Shakespeare play *Cymbeline*. This tends to confirm the attribution of this poem to Shakespeare, which remains in dispute [37]; it is especially interesting that the poem is attached to *Cymbeline*, as scholars have found overlaps in vocabulary between the poem and the play and have argued that this indicates that both were written by Shakespeare about the same time [38]. Of the two other anonymous works, the play *Famous Victories* was connected to Shakespeare, very likely because it covers exactly the same historical material as Shakespeare's *Henry V*, and *John of Bordeaux* was connected with Greene's *James IV*.

Cluster 4 consists of 35 plays and poems by 15 authors. All the poems of **Spenser** appeared as a distinct branch in this cluster. The only network of nearest neighbours in this cluster was formed by a heterogeneous combination of 11 works of which six are held to be plays by single authors, two by **Shakespeare** and two by Marlowe, in addition to one by Kyd, one by Peele, and the remaining five were of mixed or uncertain authorship. The links in this case may be genre, date, or even shared collaborative authorships. Ten of the plays are history plays, eight dealing with British history and one each with classical and French history, and the eleventh is a tragedy, *The Spanish Tragedy*. The earliest accepted dates or date ranges for the first production is 1585-9, for *The Spanish Tragedy*, and the latest 1595, for *Richard II*. This compares to the overall span of 1576 to 1642 for the set. It is likely that Shakespeare had a hand in the anonymous *Edward III* [39], and possible that Marlowe was a contributor to *Henry VI* Parts 1 and 2 [40]. Thus these are early plays, in closely related genres, with a likely overlap in collaborative authorship. It seems that in this one case these other factors were strong enough, and individual authorship was weak enough, that a clique was formed on a basis other than individual authorship.

Besides, the tree linked to this network through the play *Tancred* by Wilmot does have a more authorial character, since it includes all the Spenser poems in the set. Further, it is of interest that the set of poems by the Earl of Oxford is in this tree, and not linked to a Shakespeare poetic work or to a Shakespeare play, thus giving no support to those who argue that Shakespeare's works should be ascribed to Oxford [41].

*Cluster 5* is a single clique comprising all six plays by **Lyly** in the data set. It has no links to any other plays or network of plays. Of the other two comedies, which were not included in this clique, one, *Mother Bombie*, was connected to Shakespeare's comedy *As You Like It* in a separate tree and the second, *Woman in the Moon*, was connected to *The Maid's Tragedy*. *Woman in the Moon* is the last play Lyly wrote and is the only one of his plays in verse rather than prose. Lyly's network appeared as the most homogeneous and exclusive one in the graph, which is consistent with the idiosyncrasy of his canon, which scholars have often remarked on [42].

**Figure 4:**
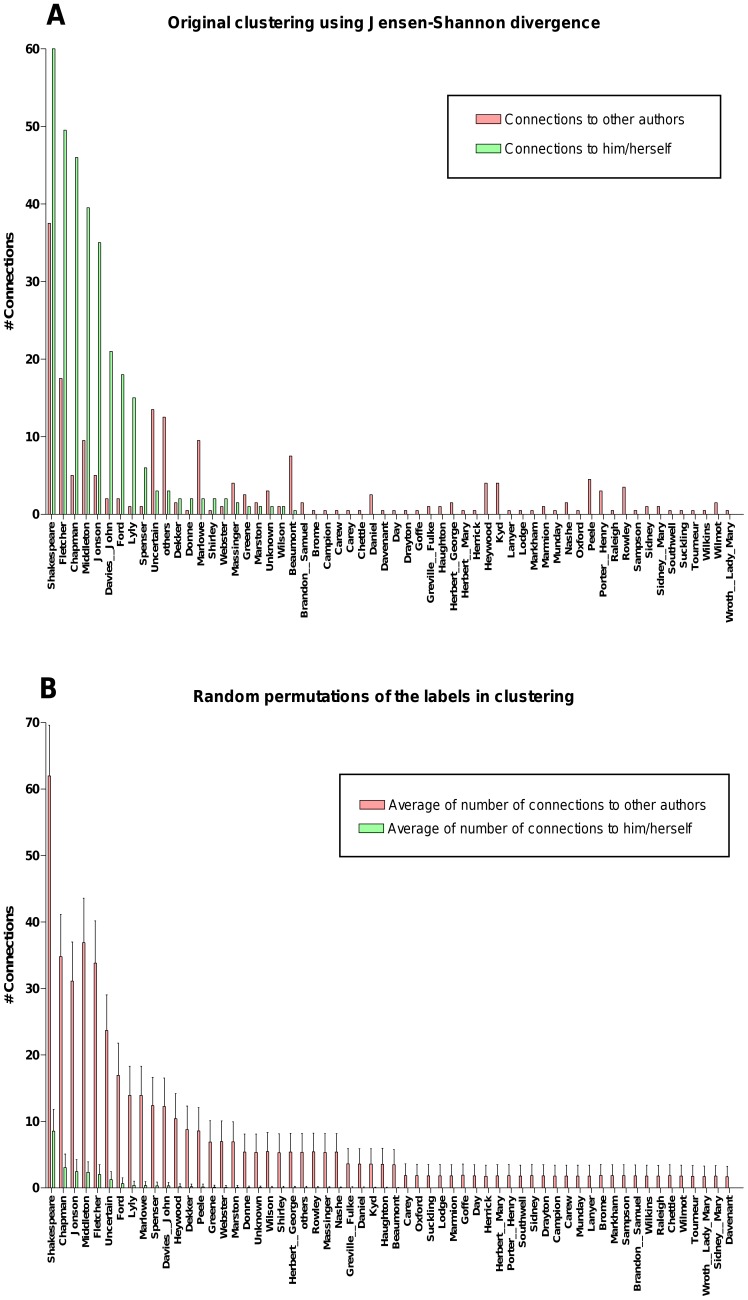
The numbers of *self* and *diff* connections observed in the Jensen-Shannon divergence based clustering and their average across 1,000 random permutations. As expected, works by major authors such as Shakespeare, Fletcher, Chapman, Middleton, Jonson, John Davies, Ford, Lyly and Spenser correlated more with their own works in the original clustering than in the randomised clusters.

To verify whether our clustering was indeed unveiling authorial affinities in the corpus, we conducted a simple permutation test on various token configurations (e.g., authors, plays/poems, genres or their combinations). In the original clustering, as expected, the tokens correlated more with their own while in the random groupings they associated more with different tokens. For example, for the authors, the observed numbers of the *self* and *diff* connections on the original and the average of 1,000 random permutations are presented in Figure 4, which demonstrates that the works by the major authors are more similar to themselves. Affinities for the combination of author + mode (plays/poems), and author + genre also appeared as highly significant for the JSD distance, suggesting that authorial affinity may be strongly captured in our results.

When testing for the influence of other metrics in the clustering, we re-clustered the data under four other metrics and performed the permutation tests. The significance of the results are presented in Table 3. From this table, it is clearly evident that among the considered metrics, the outcomes with the JSD are the most significant. Furthermore, among the considered configurations, in general, the outcomes with the “author” attribute are consistently more significant than the outcomes with other attributes or their combinations. (Additional plots for experiments for the authors are provided in File S3–File S6).

**Table 3:** Significance of authorial affinity observed on clusters obtained with different distance metrics.

Token Randomised	Metric	p-value (Wilcoxon test)	p-value (Kruskal-Wallis test 1, in observed vs. all permutations)
Authors and Plays/Poems	(JSD)	1.44948E-15	2.89844E-15
Authors and Genres	(JSD)	3.8946E-13	7.78876E-13
Authors and Plays/Poems	(Robust)	5.02E-13	1.00E-12
Authors and Genres	(Robust)	1.51E-11	3.01E-11
Authors	(JSD)	1.58834E-11	3.17643E-11
Authors	(Robust)	3.28E-10	6.56E-10
Authors and Plays/Poems	(Pearson)	2.51E-09	5.03E-09
Authors and Plays/Poems	(Cosine)	3.30E-09	6.61E-09
Authors and Genres	(Pearson)	5.89E-08	1.18E-07
Authors and Genres	(Cosine)	7.75E-08	1.55E-07
Authors	(Pearson)	1.96E-07	3.92E-07
Authors	(Cosine)	2.29E-07	4.58E-07
Authors and Plays/Poems	(Spearman)	2.65E-07	5.30E-07
Genres	(JSD)	4.26464E-06	8.52547E-06
Genres	(Spearman)	4.29E-06	8.57E-06
Genres	(Robust)	5.57E-06	1.11E-05
Authors and Genres	(Spearman)	6.34E-06	1.27E-05
Genres	(Pearson)	1.57E-05	3.14E-05
Genres	(Cosine)	1.59E-05	3.17E-05
Authors	(Spearman)	0.000123789	0.000247569
Genres and Plays/Poems	(JSD)	0.001786172	0.003571457
Genres and Plays/Poems	(Spearman)	0.004417964	0.008833922
Genres and Plays/Poems	(Robust)	0.015101187	0.030196504
Genres and Plays/Poems	(Pearson)	0.026424279	0.05283912
Genres and Plays/Poems	(Cosine)	0.026433657	0.05285789
Plays/Poems	(Spearman)	0.133101033	0.265937385
Plays/Poems	(Pearson)	0.134420845	0.268575419
Plays/Poems	(Cosine)	0.136284273	0.272299587
Plays/Poems	(JSD)	0.138705844	0.277138852
Plays/Poems	(Robust)	0.24252702	0.484669997

In addition to Jensen-Shannon divergence, we utilised Spearman's, Pearson's, cosine and a robust metric (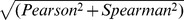) to produce the distance matrix and re-perform the clustering. On that our randomisation process resulted in *p-values* for each metric and token configuration as shown in the table. For all metrics, the p-values associated with the **Kruskal-Wallis test 2** in observed vs. each permutation were highly significant (close to zero, not shown). Further, the results for the JSD has been the most significant in terms of authorial affinities.

**Figure 5:**
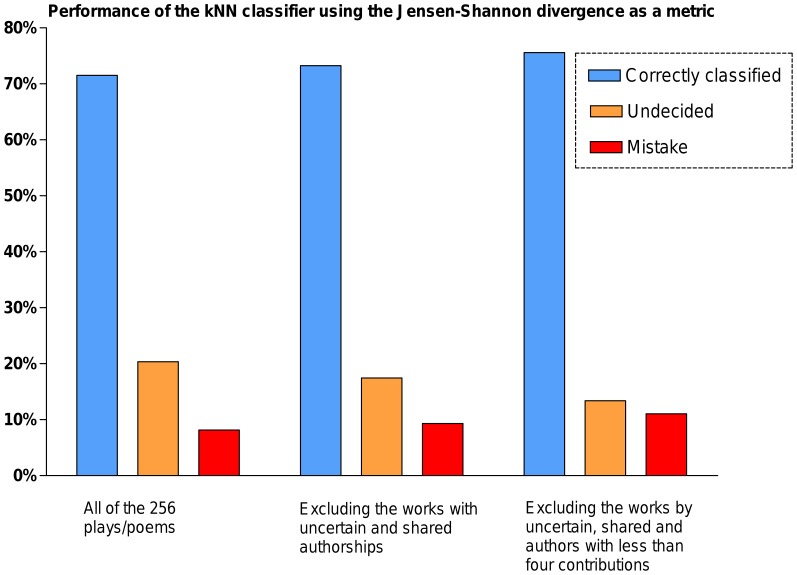
The kNN classification (for *k* = 3) using the Jensen-Shannon divergence as a metric. The JSD successfully classified the authorship of 71.51% of the plays/poems. Further, when we removed the unknown, uncertain and shared works, it classified 73.25% of all works which was further increased to 75.58% by removing the works with authors having less than four contributions.

Finally, when we conducted the kNN classification test (for *k* = 3) using the JSD as a metric, we successfully classified the authorship of 71.51% of the plays/poems of authors that have more than three contributions in the dataset. By removing the unknown, uncertain and shared authored works, the classification performance becomes 75.58% of works from authors having more than three contributions; the results are presented in Figure 5. Besides, a kNN classification of the disputed works (Table 4) further evidenced the previous JSD cluster based authorship assignment in Table 2. The computational steps for this test are given in File S7.

**Table 4:** Information Theory based kNN classification of the works of uncertain authorship.

Uncertain works	Authorship based on the kNN classifier	Remark/nearest neighbours
Bloody Brother	Fletcher	-
Ieronimo	Undecided	Beaumont and Fletcher, Middleton, Kyd
Warning for Fair Women	Undecided	Porter Henry, Ford, Heywood
Arden	Undecided	Uncertain (Warning for Fair Women), Porter Henry, Beaumont and Fletcher. As a further note, Haughton, Heywood and Shakespeare appeared as the 4 to 6 nearest neighbours, respectively
John of Bordeaux	Undecided	Greene, Haughton, Uncertain(Warning for Fair Women)
Fair Em	Undecided	Uncertain (King Leir), Haughton, Shakespeare
King Leir	Undecided	Shakespeare, Beaumont and Fletcher, Haughton
Knack to Know a Knave	Undecided	Uncertain (King Leir), Shakespeare, Chapman
Lovers Complaint	Shakespeare	-
Famous Victories	Shakespeare	-
Soliman and Perseda	Undecided	Kyd, Shakespeare and others, Shakespeare
Selimus Part 1	Shakespeare and others	-
Wars of Cyrus	Shakespeare and others	-
Edward III	Shakespeare and others	-
Edmond Ironside	Shakespeare and others (weak classification)	Shakespeare, Uncertain (King Leir), Shakespeare and others
Troublesome Reign King John I	Shakespeare and others (weak classification)	Shakespeare, Unknown Troublesome Reign King John II), Shakespeare and others
Troublesome Reign King John II	Shakespeare and others (weak classification)	Unknown(Troublesome Reign King John I), Shakespeare and others, Shakespeare

The authorship is determined by majority voting in the kNN (for *k* = 3) computed using the JSD. A weak classification is noted when the work is voted by the same but in conjunction with “other” authors.

**Figure 6:**
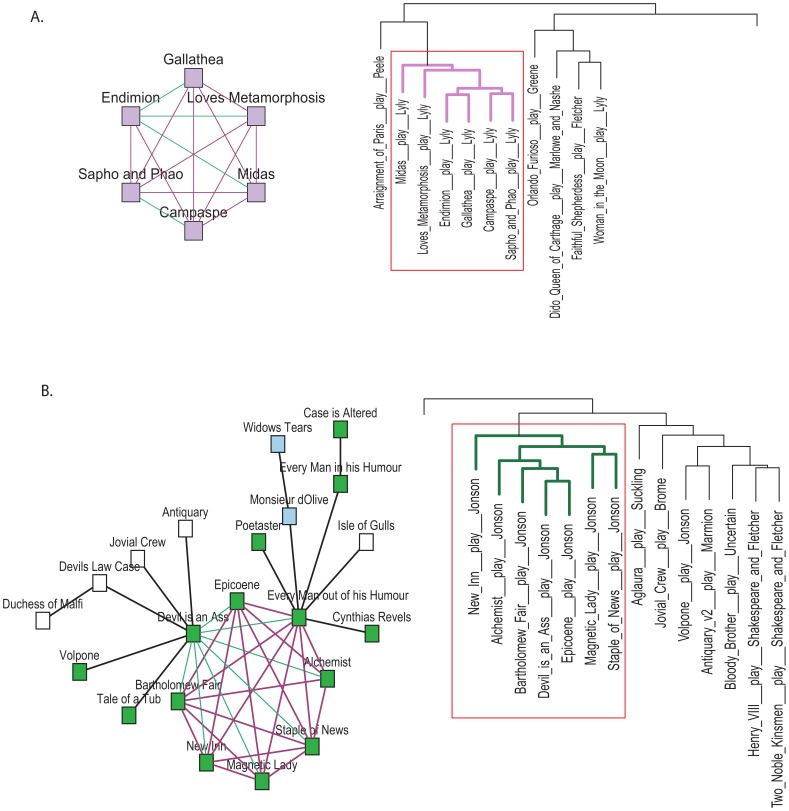
Comparisons between partial kNN paracliques and hierarchical clustering outcomes. The paraclique structures formed by Lyly and Jonson appeared as two separate branches in the hierarchical clustering. Both methods utilise the Jensen-Shannon divergence as a metric.

The proposed method in conjunction with the JSD provides a natural solution in finding authorship affinities via a reasonable balance between parametric and non-parametric optimization criteria. This became evident when we applied two other widely known clustering methods using R: hierarchical clustering (stats::hclust, see results in File S8) and K-Means (cluster::pam, silhouette, for *K* = 3 to 60, see results in File S9) on the same distance matrix. The outcomes were highly comparable against our previous results both in terms of authorship and genre affinities. For instance, as shown in Figure 6, the paraclique structures formed by Lyly and Jonson appeared as two separate branches in the hierarchical clustering. However, the mixture of authorship persisted in both methods. In the the K-Means clustering, as the value of K is increased, the segregation of individual works from larger groups becomes more evident without improving the authorship homogeneity found in our paraclique structures. This indicates that our method, while producing comparable outcomes, provides an additional instrument via the kNN structures to precisely investigate the authorship and/or genre affinities independent of assumptions about the number of authors.

## Conclusions and Future Work

Our clustering process produced an astonishing predominance of authorial affinities in the corpus. The mode of the work (non-dramatic poetry versus play) also becomes clearly differentiated in our clustering. Our results show that our clustering approach, in conjunction with the JSD, provides a soundly based guide to the authorship of plays and poems where attribution is unknown or disputed. However, such authorship attribution should be further investigated by other methods. While this work focuses on applications in language-based research, our analytical approach is not domain-specific and could feasibly be applied to the analysis of large data sets in other domain areas; for example in a biological setting, patient classification using gene co-expressions.

## Supporting Information

File S1 **Complete text corpus dataset (i.e. the frequencies of 66,907 unique words in the 256 texts).**
(TXT)

File S2 **A distance matrix (256×256) based on Jensen-Shannon divergence.**
(TXT)

File S3 **Author to work associations using Cosine distance.**
(TIF)

File S4 **Author to work associations using a robust correlation.**
(TIF)

File S5 **Author to work associations using Pearson's correlation.**
(TIF)

File S6 **Author to work associations using Spearman's rank computation.**
(TIF)

File S7 **The kNN classification (** ***k*** **= 3) of the authors using the JSD as a metric.**
(XLSX)

File S8 **Hierarchical clustering of the works using the JSD as a metric.**
(PDF)

File S9 **K-Means clustering of the works using the JSD as a metric.**
(PDF)
