# Supplementary material for: An Information Theoretic Clustering Approach for Unveiling Authorship Affinities in Shakespearean Era Plays and Poems
Source: PLoS One. 2014 Oct 27;9(10):e111445. doi: 10.1371/journal.pone.0111445 (PMC4210181; doi:10.1371/journal.pone.0111445)

K-Means clustering using JSD as a metric (File S9)

K-Means Silhouette

K	Max silhouette	Min silhouette	Avg silhouette	Median silhouette	SD silhouette
**3**	0.141249	-0.09691	0.01028	-0.00062	0.057646
**4**	0.103918	-0.09033	0.00579	0.001644	0.033285
**5**	0.08759	-0.09336	-0.0004	-0.0001	0.033229
**6**	0.082057	-0.07874	0.000245	0.001184	0.033786
**7**	0.120895	-0.09818	-0.00444	-0.00609	0.045686
**8**	0.120895	-0.10985	-0.00172	-0.00426	0.044932
**9**	0.13107	-0.08058	0.0025	-0.00195	0.042987
**10**	0.13107	-0.08058	0.003749	0.000333	0.042591
**11**	0.13107	-0.08138	0.005374	0.000895	0.037716
**12**	0.13107	-0.08138	0.005741	8.69E-05	0.038527
**13**	0.13107	-0.07063	0.005918	0	0.037756
**14**	0.143011	-0.09187	0.007357	0.003579	0.043728
**15**	0.143011	-0.09187	0.007382	0.00316	0.043621
**16**	0.143011	-0.09187	0.007756	0.00316	0.0431
**17**	0.143965	-0.07546	0.010633	0.003947	0.041135
**18**	0.143965	-0.07546	0.011154	0.004963	0.040367
**19**	0.143965	-0.07546	0.012238	0.005434	0.042012
**20**	0.143965	-0.07546	0.012508	0.006872	0.041685
**21**	0.143965	-0.07546	0.012404	0.004944	0.043734
**22**	0.143965	-0.07546	0.01272	0.004944	0.043477
**23**	0.143965	-0.07546	0.01255	0.004899	0.043491
**24**	0.143965	-0.07546	0.012667	0.005071	0.043373
**25**	0.143965	-0.07546	0.013634	0.008142	0.041571
**26**	0.143965	-0.07546	0.01371	0.008026	0.041569
**27**	0.143965	-0.07546	0.01442	0.008347	0.041477
**28**	0.143965	-0.07546	0.014826	0.008994	0.041209
**29**	0.143965	-0.07546	0.014572	0.008345	0.041055
**30**	0.143965	-0.07546	0.014989	0.008528	0.040773
**31**	0.143965	-0.07546	0.015305	0.008675	0.040629
**32**	0.143965	-0.07546	0.015706	0.009441	0.040305
**33**	0.143965	-0.07546	0.015656	0.008994	0.040566
**34**	0.143965	-0.07546	0.015998	0.008675	0.040626
**35**	0.143965	-0.07546	0.01587	0.008528	0.040869
**36**	0.143965	-0.07546	0.016301	0.009686	0.040602
**37**	0.143965	-0.06892	0.017077	0.009686	0.039608
**38**	0.143965	-0.06892	0.01703	0.008994	0.039718

**39**	0.143965	-0.06892	0.017308	0.009686	0.039601
**40**	0.141686	-0.06702	0.017391	0.0093	0.039119
**41**	0.141686	-0.06702	0.017266	0.009075	0.0389
**42**	0.139114	-0.08095	0.018012	0.01336	0.040741
**43**	0.136873	-0.07986	0.018225	0.012173	0.040404
**44**	0.136873	-0.07491	0.018762	0.012173	0.039666
**45**	0.136873	-0.07491	0.019863	0.012976	0.040143
**46**	0.136873	-0.07491	0.019847	0.012976	0.040075
**47**	0.136873	-0.07491	0.019998	0.013673	0.040025
**48**	0.136873	-0.07491	0.020012	0.013157	0.04
**49**	0.136873	-0.07491	0.020098	0.013157	0.040028
**50**	0.136873	-0.07491	0.01973	0.012602	0.039997
**51**	0.136873	-0.07491	0.019708	0.012173	0.040029
**52**	0.136873	-0.07094	0.02078	0.013708	0.039687
**53**	0.136873	-0.07094	0.020476	0.01444	0.03925
**54**	0.136873	-0.07094	0.020583	0.013708	0.03948
**55**	0.136873	-0.07094	0.020765	0.012717	0.039905
**56**	0.136873	-0.06645	0.021201	0.012717	0.039326
**57**	0.136873	-0.06645	0.020348	0.012173	0.038691
**58**	0.136873	-0.06645	0.020443	0.011601	0.039075
**59**	0.136873	-0.06645	0.020182	0.011427	0.039085
**60**	0.136873	-0.06105	0.020761	0.011427	0.03837

Silhouette values have been computed using *silhouette()* function in R, according to K clusters.

(For each observation i, the *silhouette width* s(i) is defined as follows: Put a(i) = average dissimilarity between i and all other points of the cluster to which i belongs (if i is the *only* observation in its cluster, s(i) := 0 without further calculations). For all *other* clusters C, put d(i,C) = average dissimilarity of i to all observations of C. The smallest of these d(i,C) is b(i) := \min_C d(i,C), and can be seen as the dissimilarity between i and its “neighbor” cluster, i.e., the nearest one to which it does *not* belong. Finally, s(i) := (b(i) - a(i)) / max(a(i), b(i))).

Color legends

Author	Color	Author	Color
**Beaumont**	#FFFFFF	**Marlowe**	#FFFFFF
**Beaumont_and_Fletcher**	#99CC00	**Marlowe_and_Nashe**	#FFFFFF
**Brandon_Samuel**	#FFFFFF	**Marmion**	#FFFFFF
**Brome**	#FFFFFF	**Marston**	#FFFFFF
**Campion**	#FFFFFF	**Massinger**	#FFFFFF
**Carew**	#FFFFFF	**Middleton**	#99CCFF
**Carey**	#FFFFFF	**Middleton_and_Rowley**	#99CCFF
**Chapman**	#FF6600	**Munday**	#FFFFFF
**Chettle**	#FFFFFF	**Nashe**	#FFFFFF
**Daniel**	#FFFFFF	**Oxford**	#FFFFFF
**Davenant**	#FFFFFF	**Peele**	#FFFFFF
**Davies_John**	#FFFFFF	**Porter_Henry**	#FFFFFF
**Day**	#FFFFFF	**Raleigh**	#FFFFFF
**Dekker**	#FFFFFF	**Rowley**	#FFFFFF
**Donne**	#FFFFFF	**Shakespeare**	#CC99FF
**Drayton**	#FFFFFF	**Shakespeare_and_Fletcher**	#CC99FF
**Fletcher**	#99CC00	**Shakespeare_and_Middleton**	#CC99FF
**Fletcher_and_Massinger**	#99CC00	**Shakespeare_and_Wilkins**	#CC99FF
**Ford**	#FFFFFF	**Shakespeare_and_others**	#CC99FF
**Goffe**	#FFFFFF	**Shirley**	#FFFFFF
**Greene**	#FFFFFF	**Sidney**	#FFFFFF
**Greville_Fulke**	#FFFFFF	**Sidney_Mary**	#FFFFFF
**Haughton**	#FFFFFF	**Southwell**	#FFFFFF
**Herbert_George**	#FFFFFF	**Spenser**	#FFFFFF
**Herbert_Mary**	#FFFFFF	**Suckling**	#FFFFFF
**Herrick**	#FFFFFF	**Tourneur**	#FFFFFF
**Heywood**	#FFFFFF	**Uncertain**	#FF0000
**Jonson**	#008000	**Unknown**	#FF0000
**Kyd**	#FFFFFF	**Webster**	#FFFFFF
**Lanyer**	#FFFFFF	**Wilmot**	#FFFFFF
**Lodge**	#FFFFFF	**Wilson**	#FFFFFF
**Lyly**	#FFFFFF	**Wroth_Lady Mary**	#FFFFFF
**Markham_and_Sampson**	#FFFFFF		

Graphical representations of K-Means clustering (for K = 3 to 60)

Original

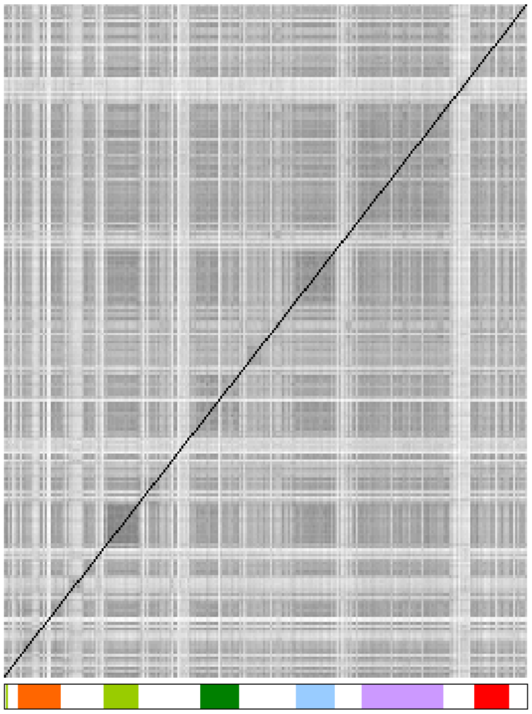

3 Clusters

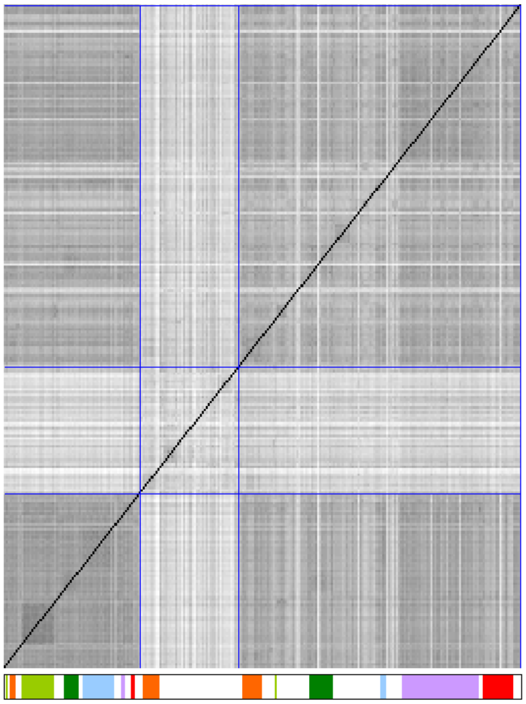

4 Clusters

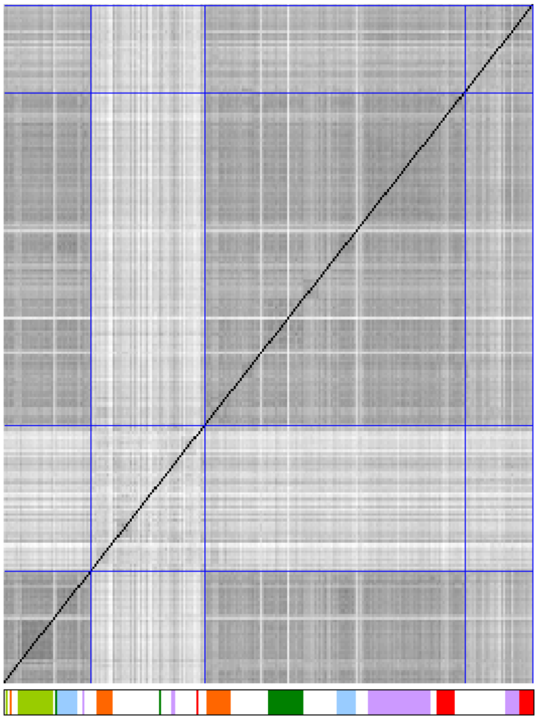

5 Clusters

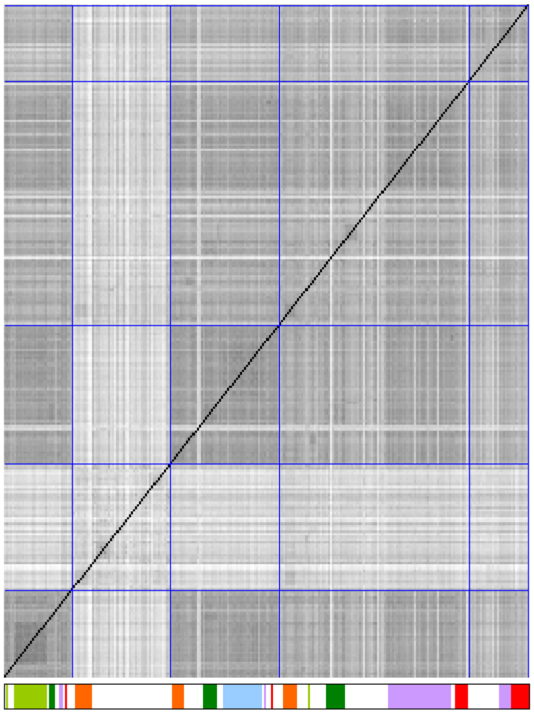

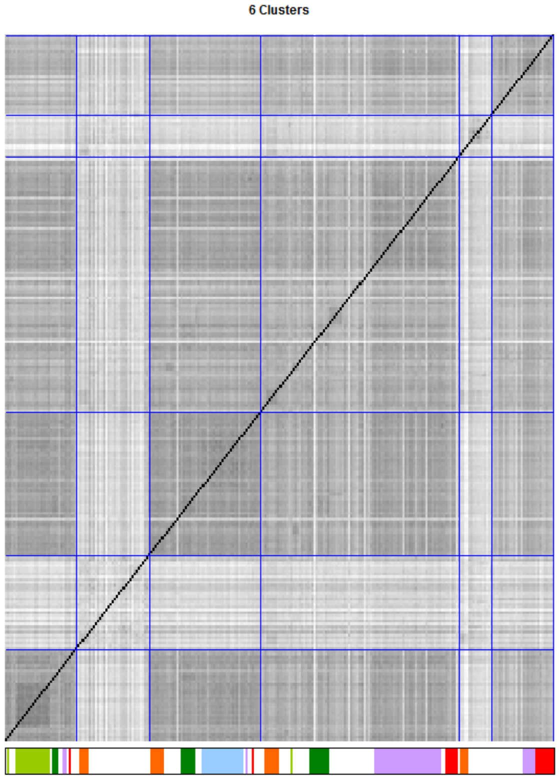
6 Clusters

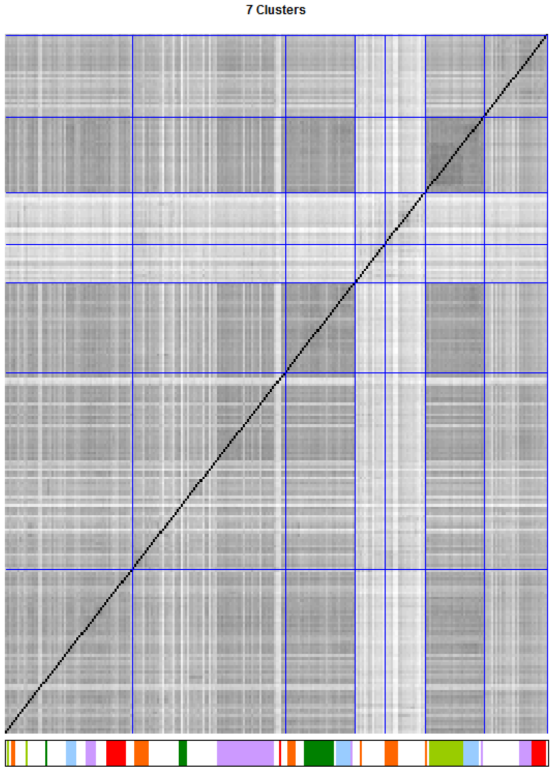
7 Clusters

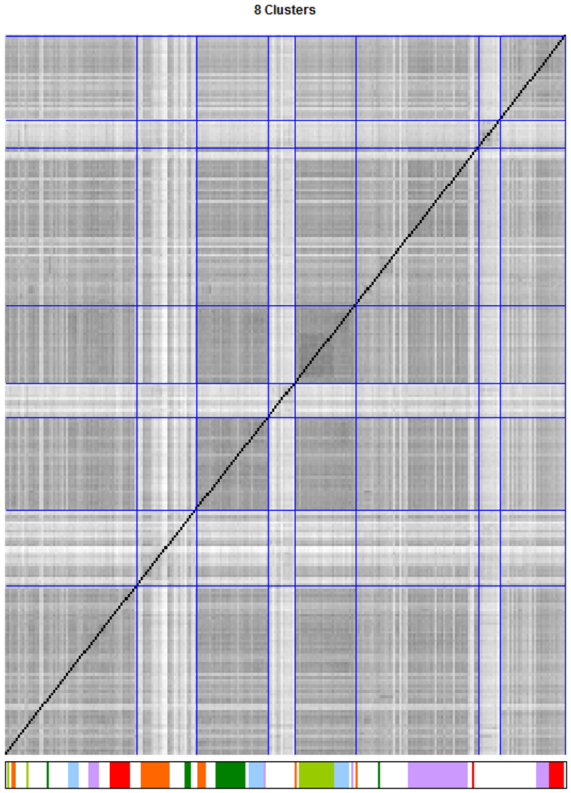
8 Clusters

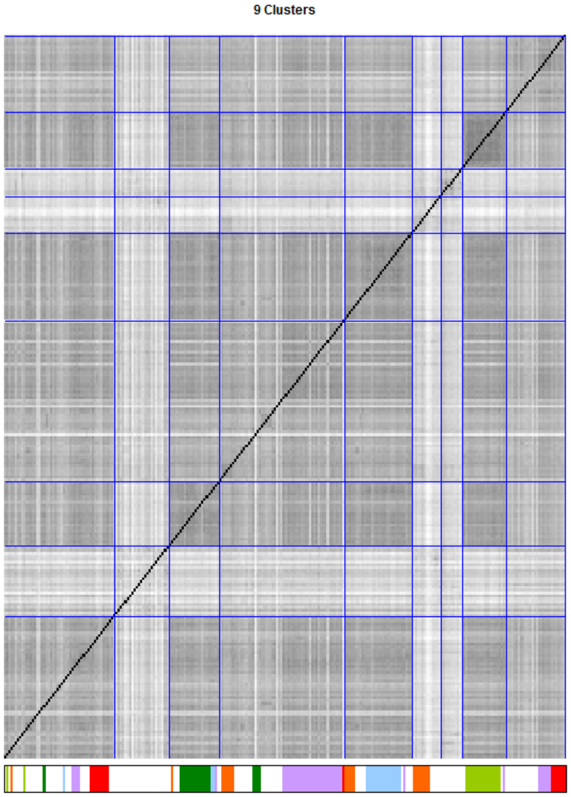
9 Clusters

10 Clusters

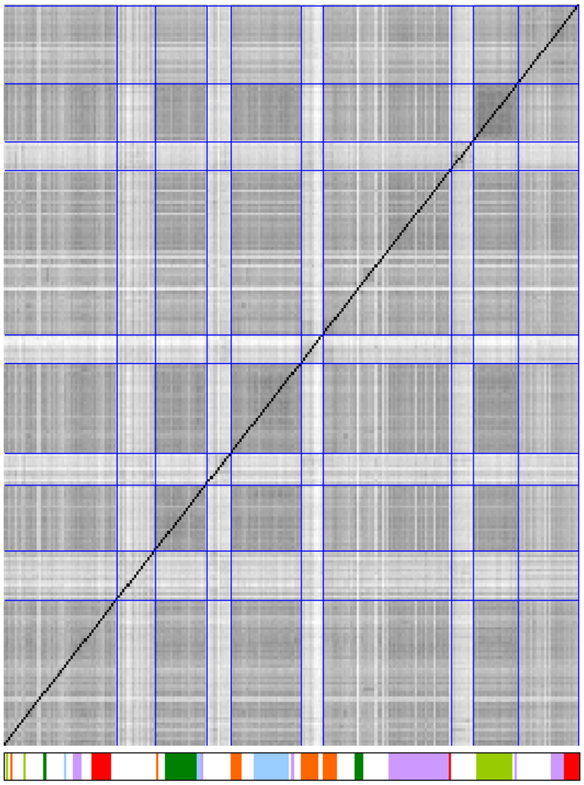

11 Clusters

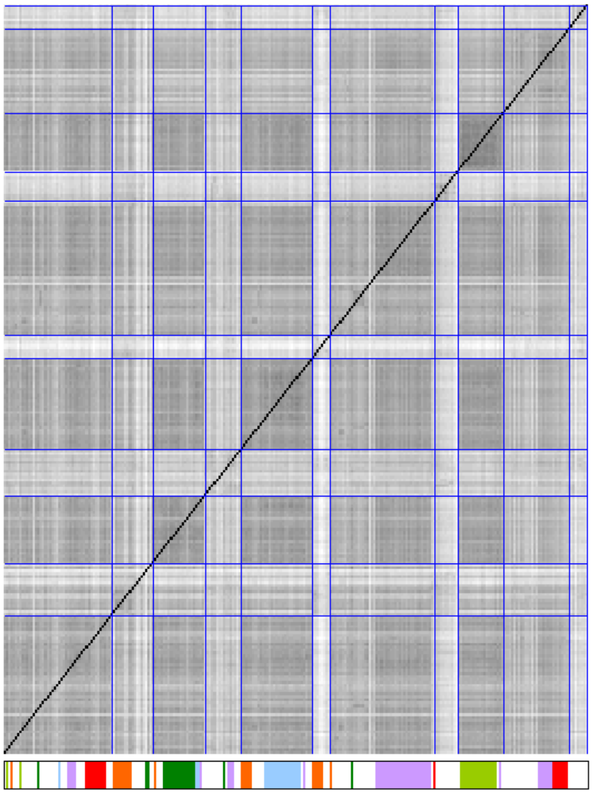

12 Clusters

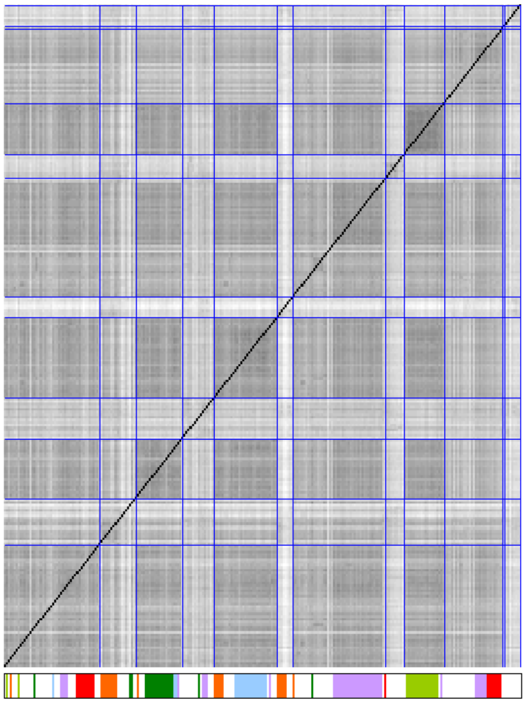

13 Clusters

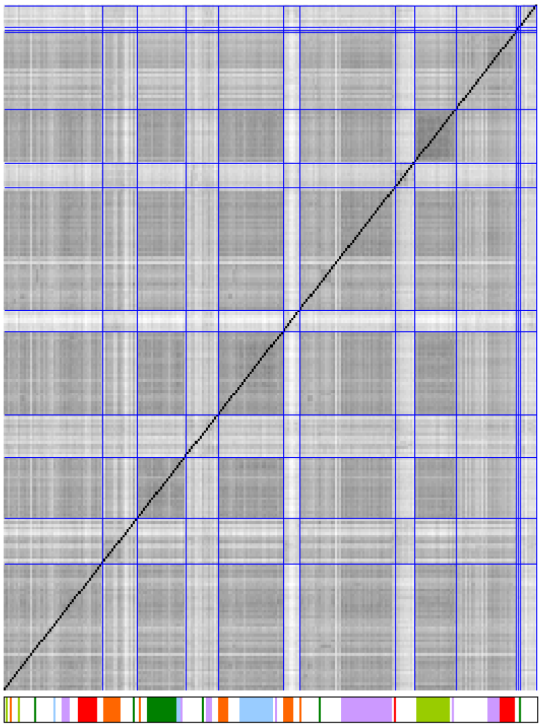

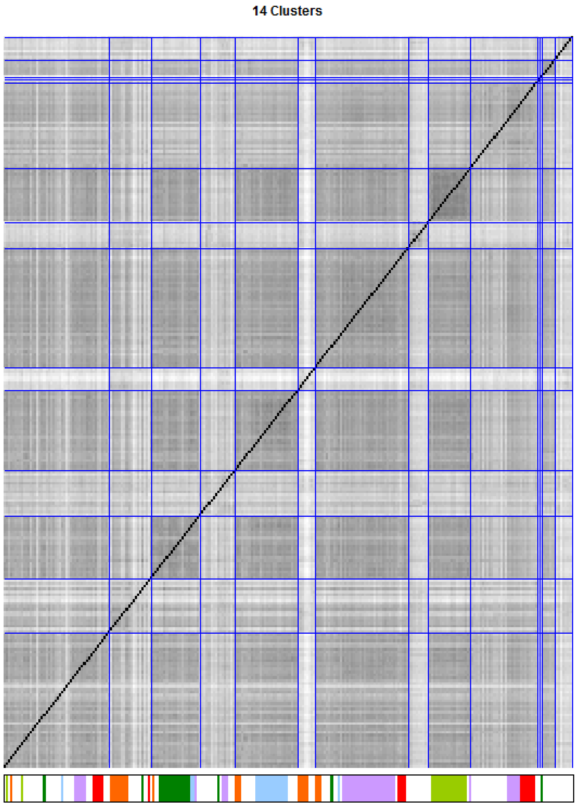
14 Clusters

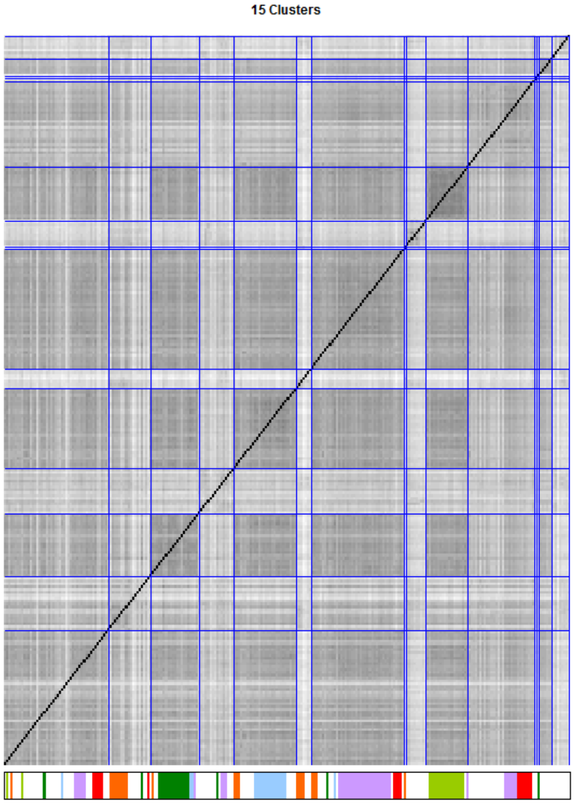
15 Clusters

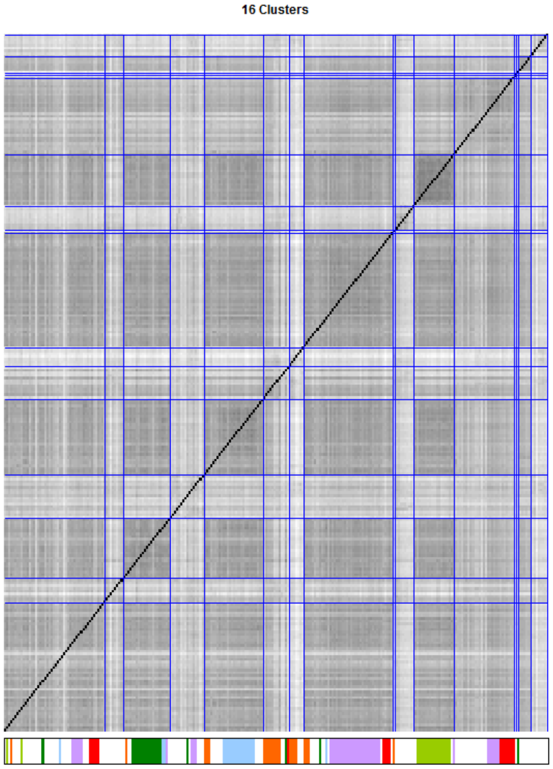
16 Clusters

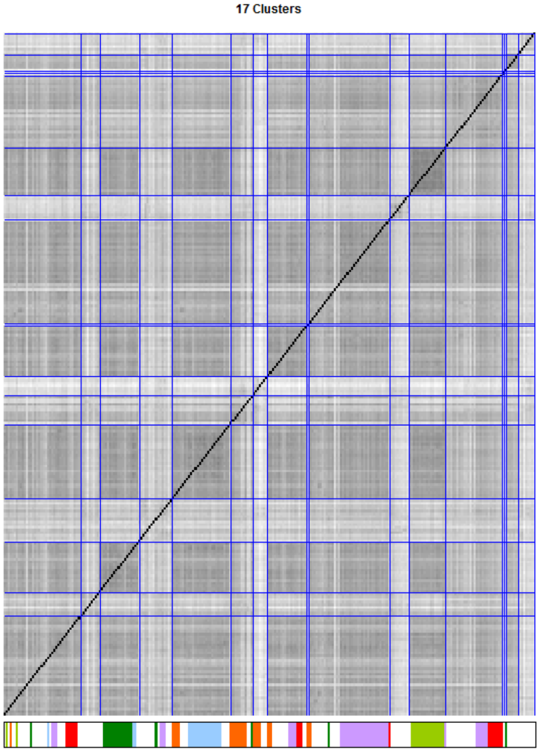
17 Clusters

18 Clusters

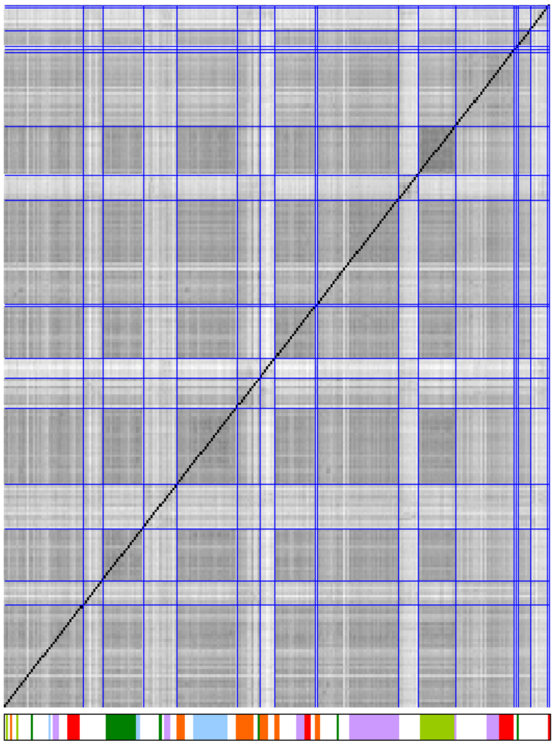

19 Clusters

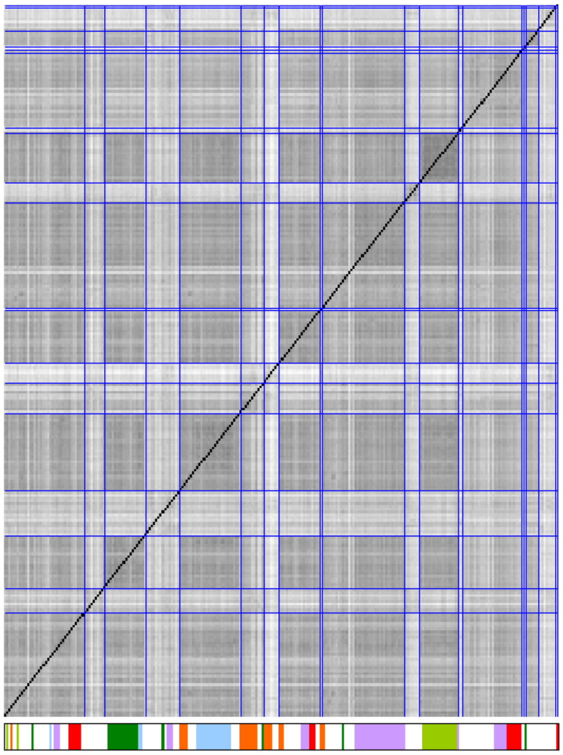

20 Clusters

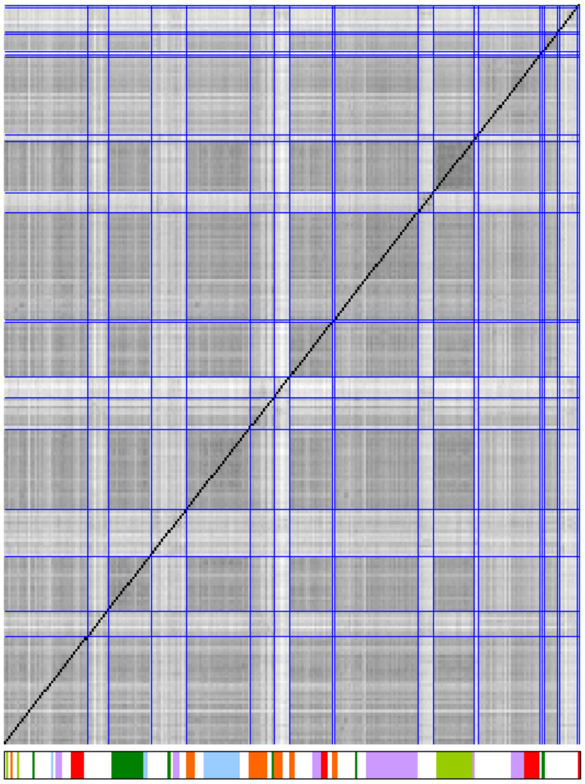

21 Clusters

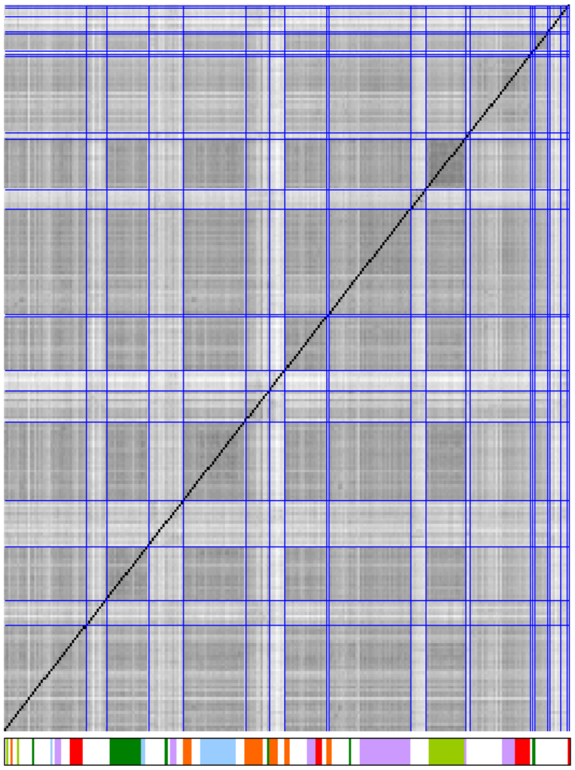

22 Clusters

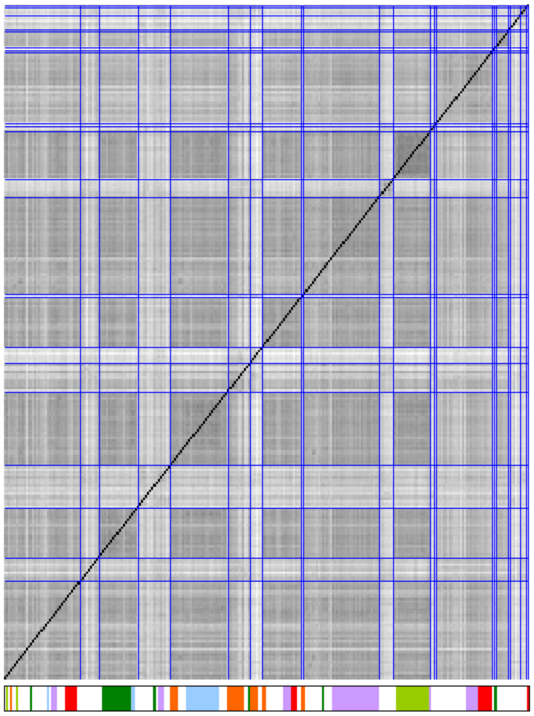

23 Clusters

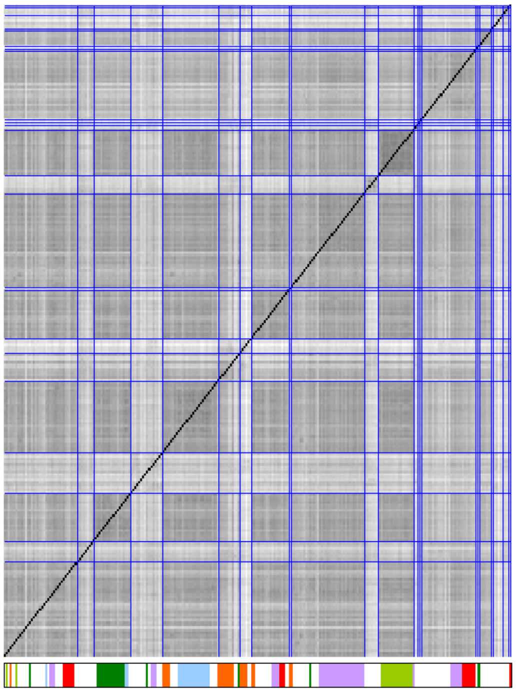

24 Clusters

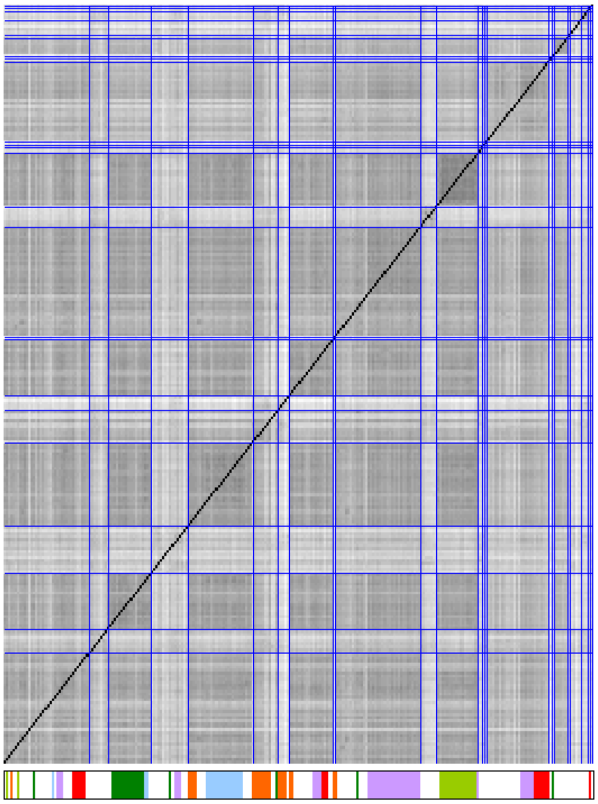

25 Clusters

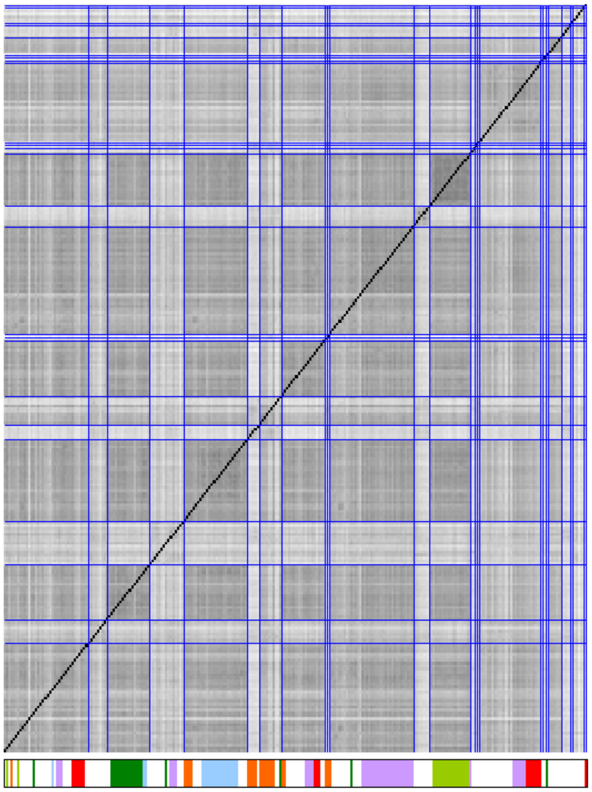

26 Clusters

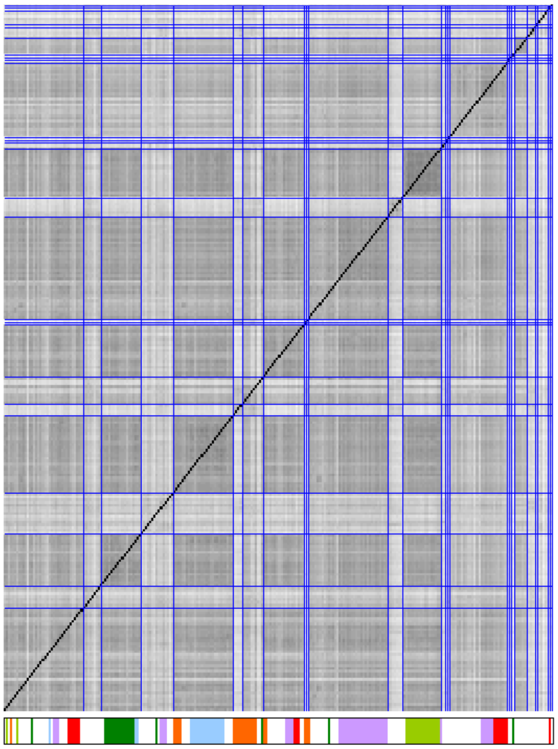

27 Clusters

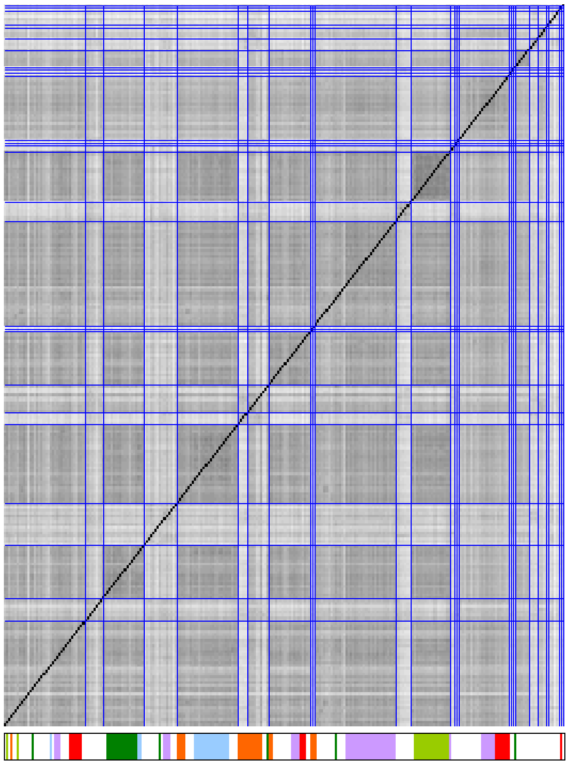

28 Clusters

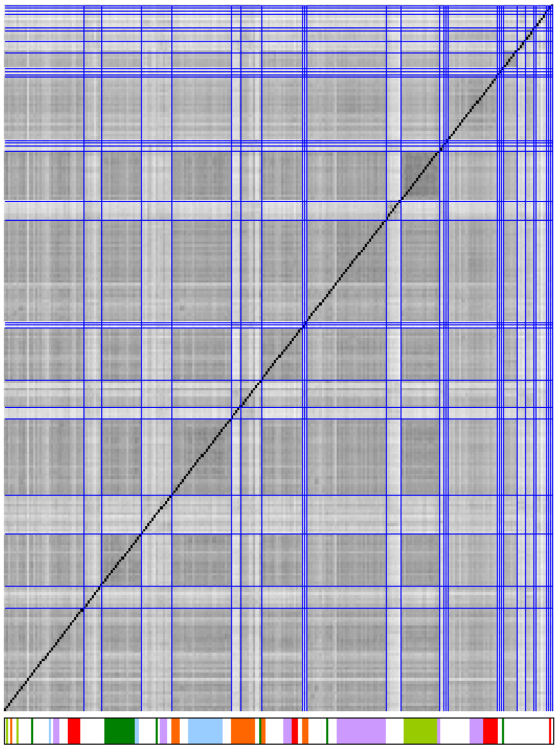

29 Clusters

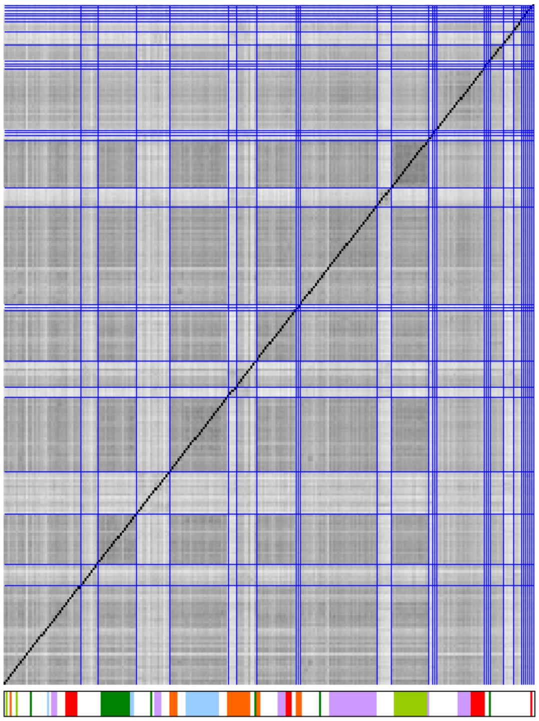

30 Clusters

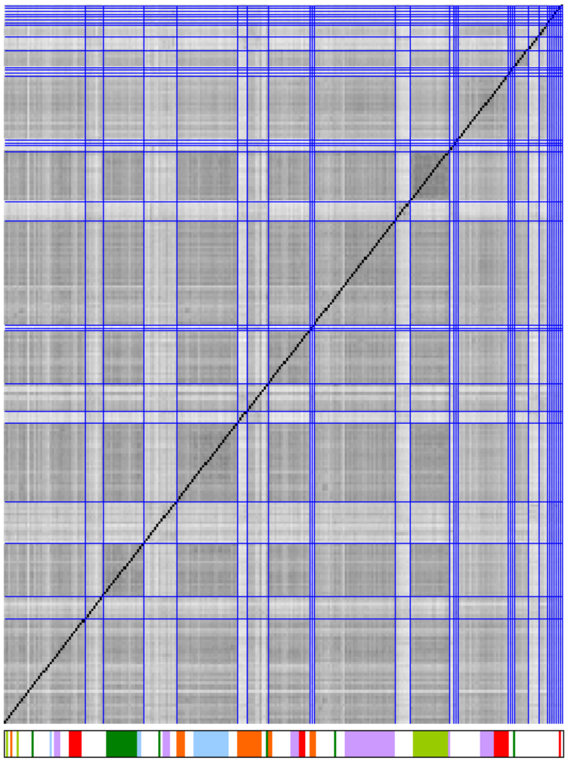

31 Clusters

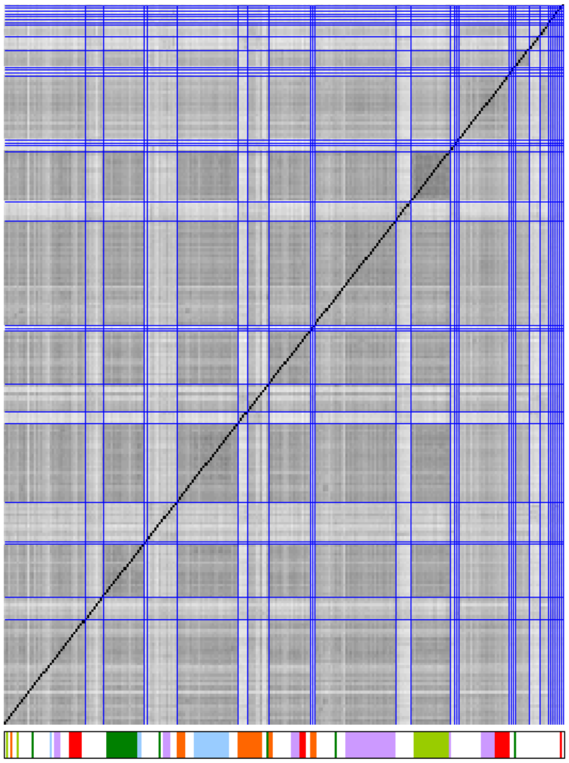

32 Clusters

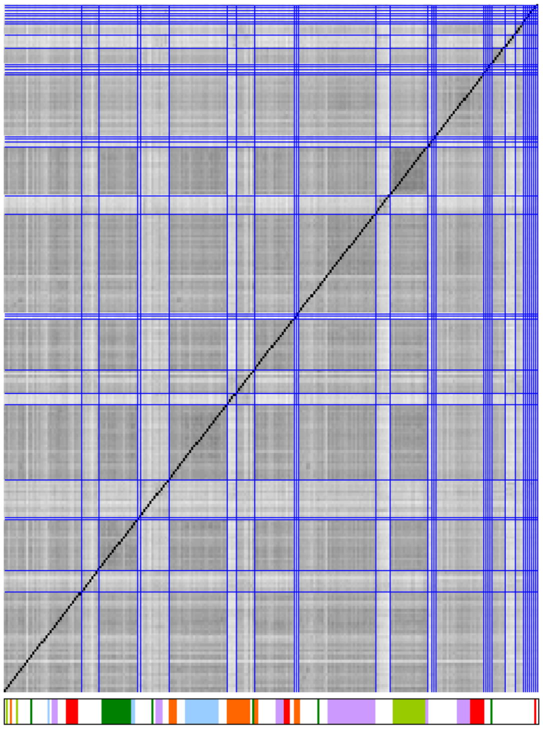

33 Clusters

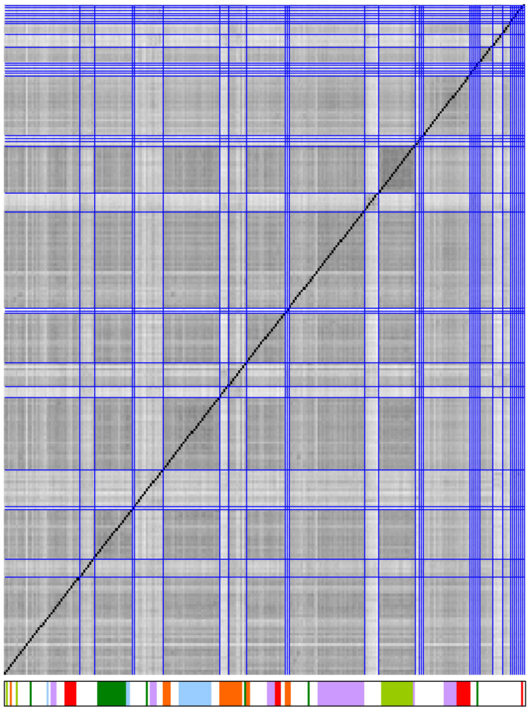

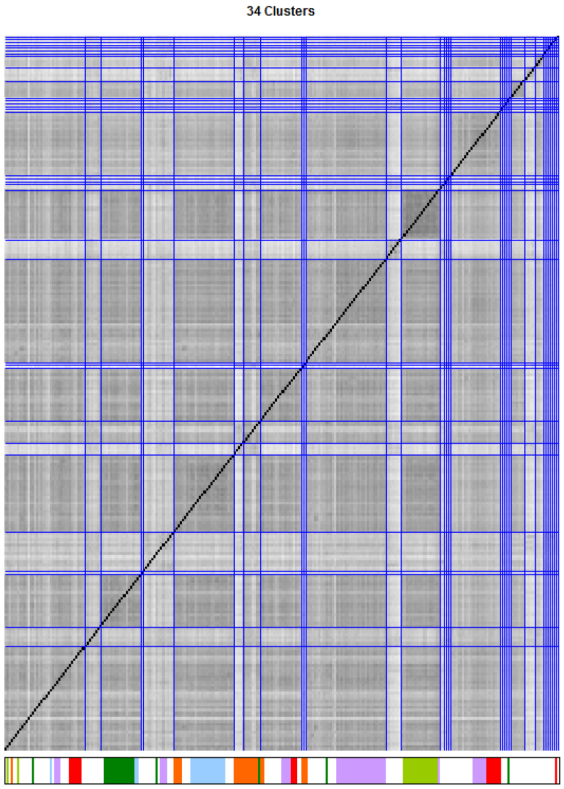
34 Clusters

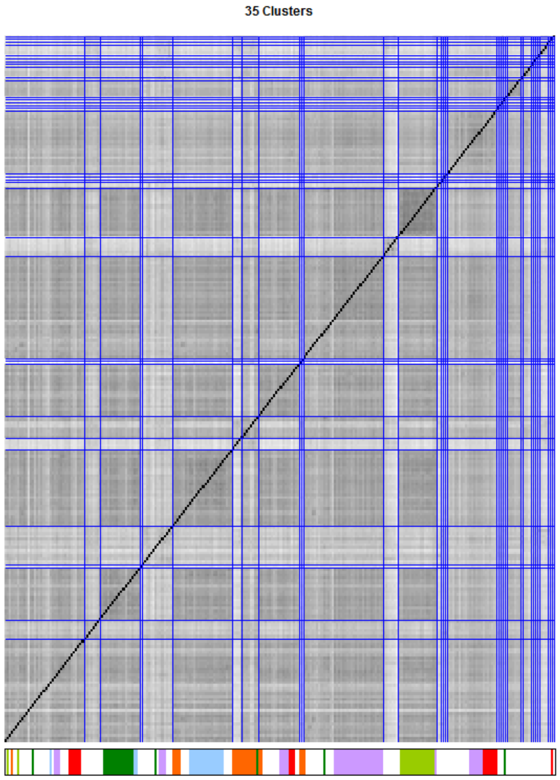
35 Clusters

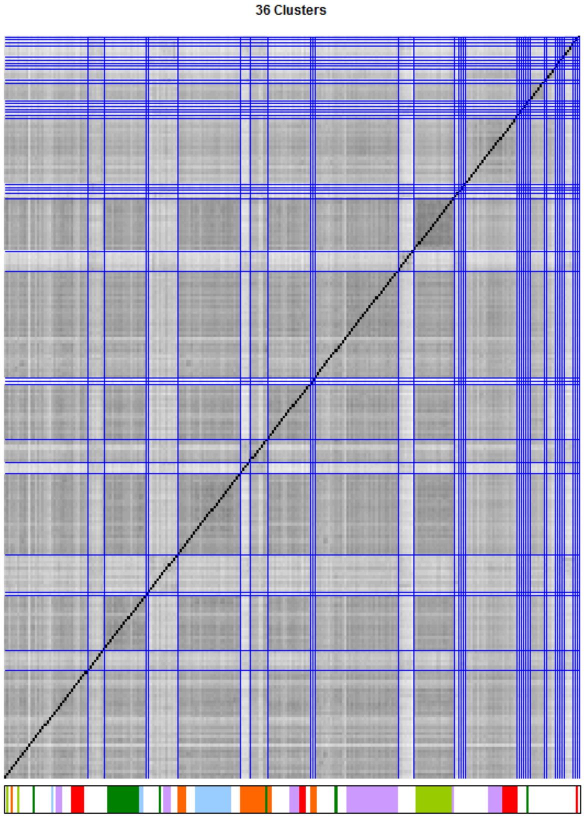
36 Clusters

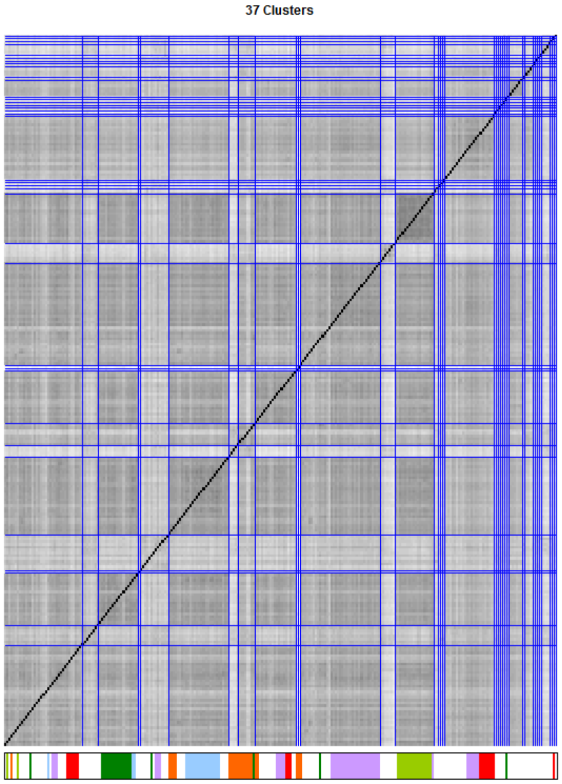
37 Clusters

38 Clusters

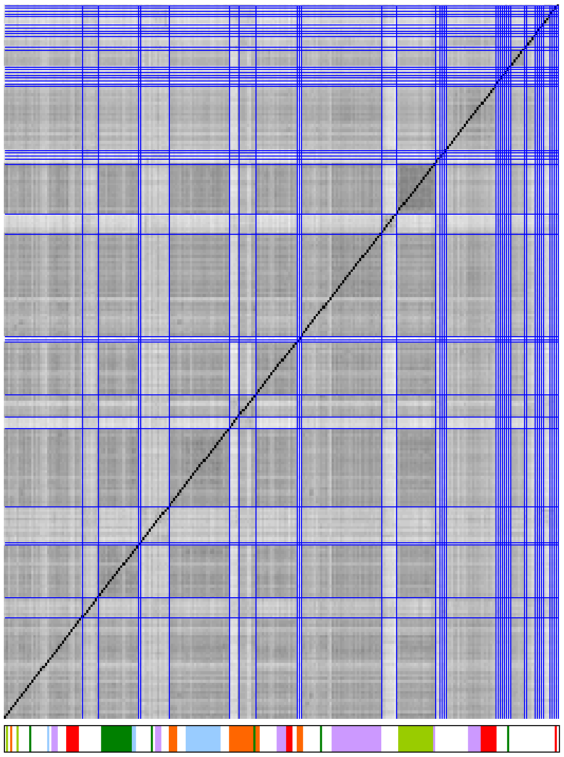

39 Clusters

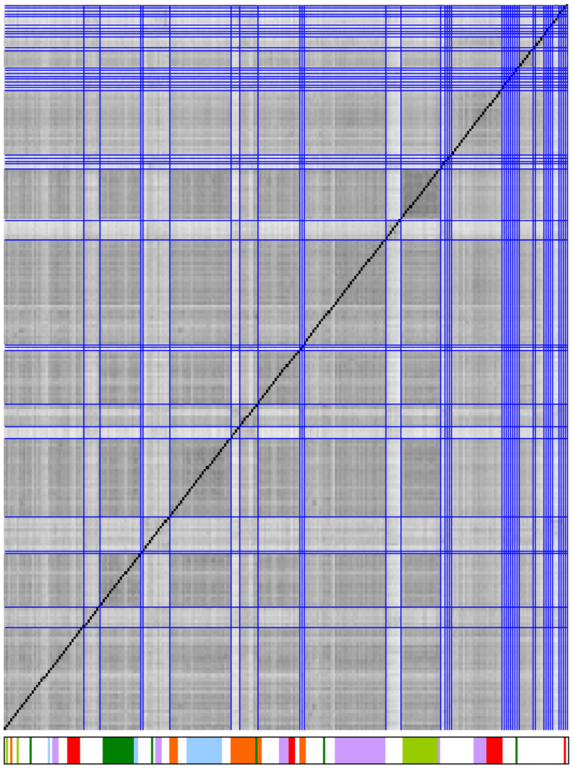

40 Clusters

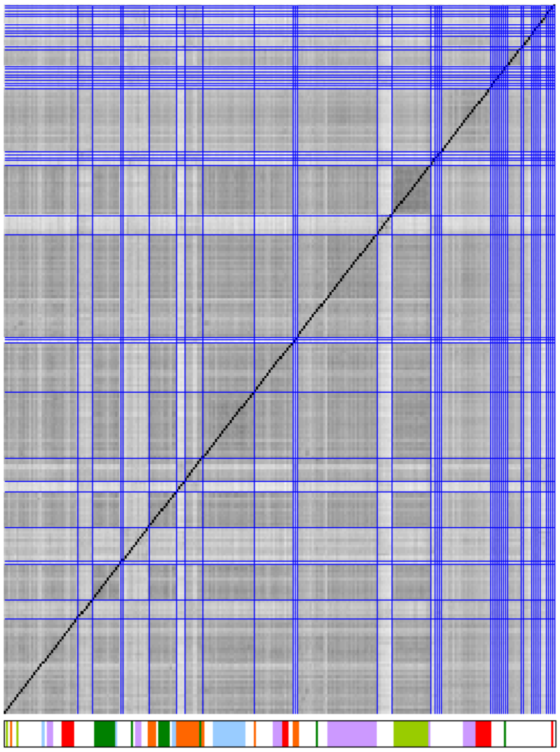

41 Clusters

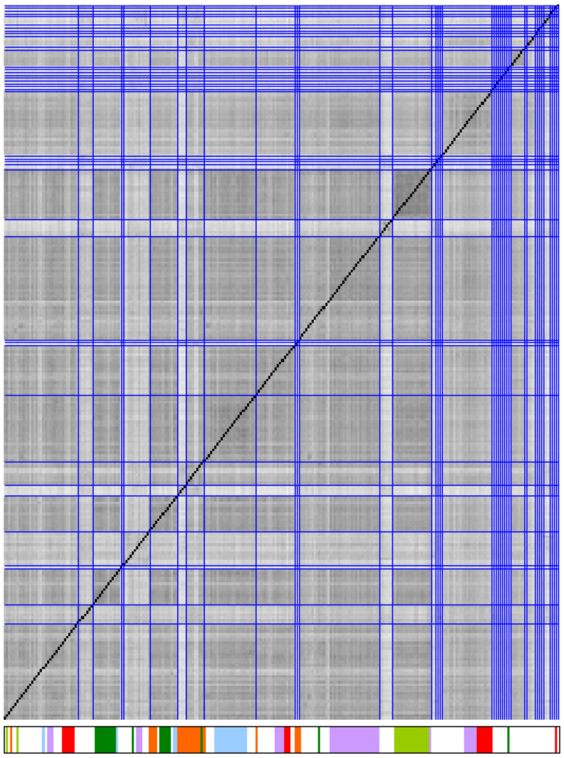

42 Clusters

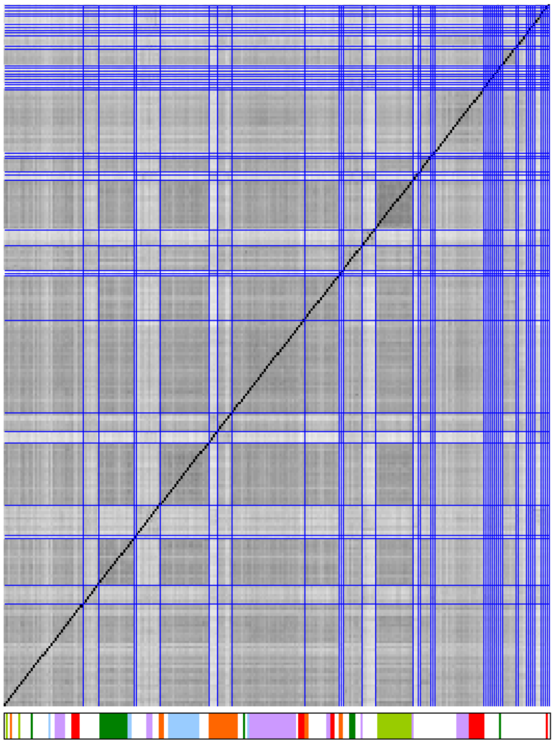

43 Clusters

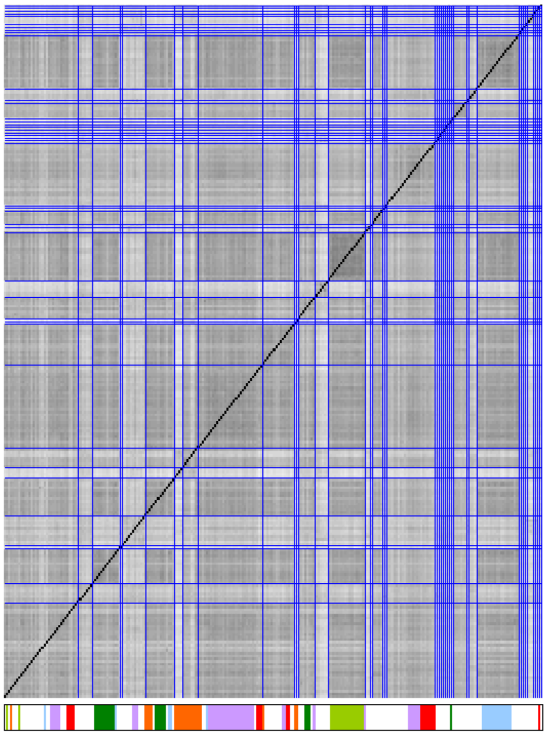

44 Clusters

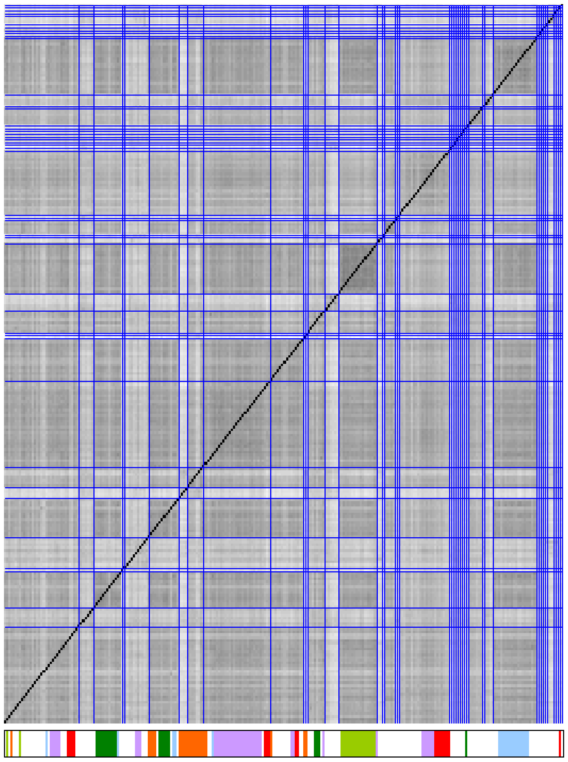

45 Clusters

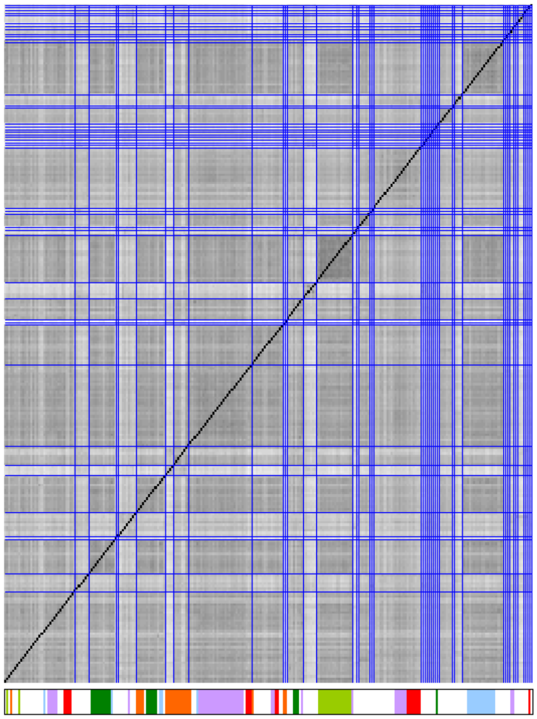

46 Clusters

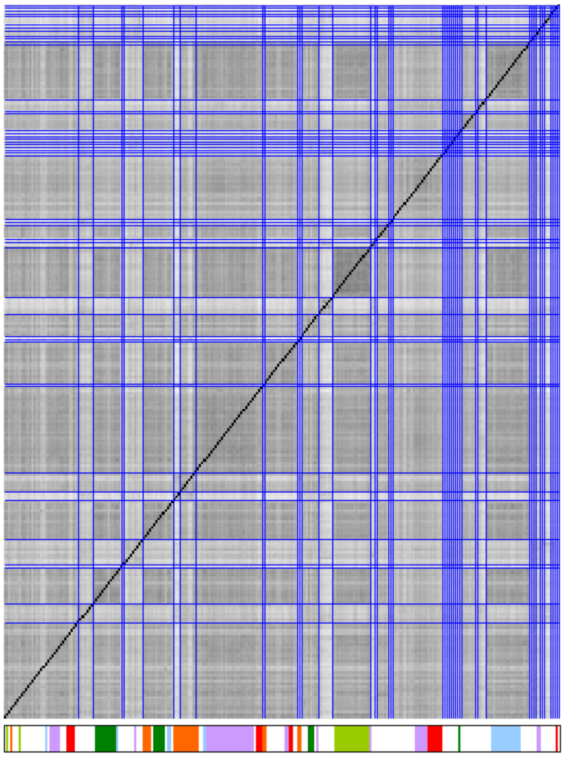

47 Clusters

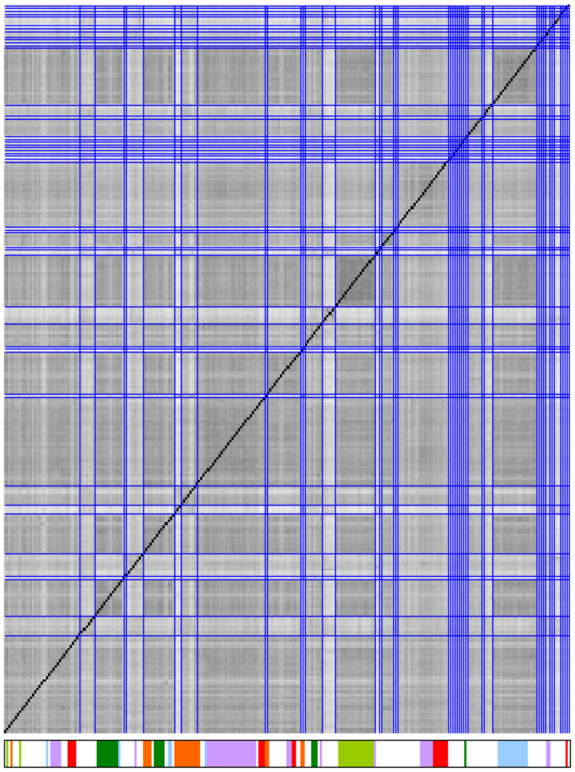

48 Clusters

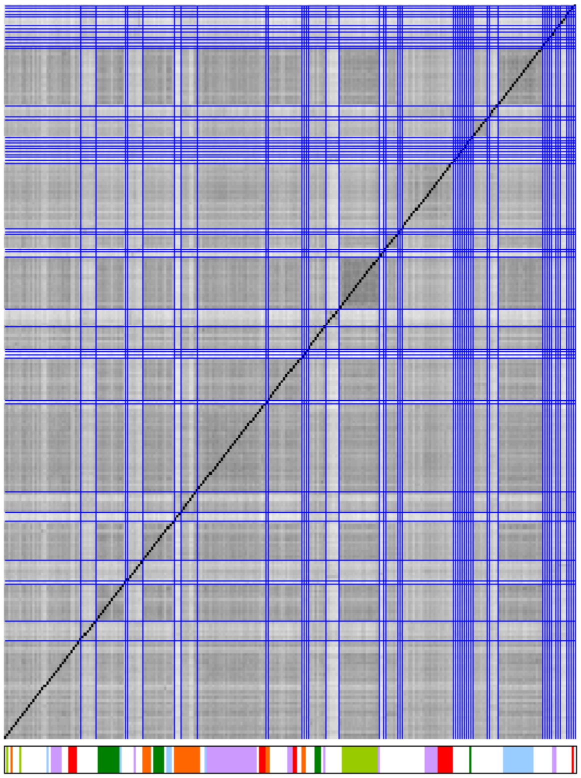

49 Clusters

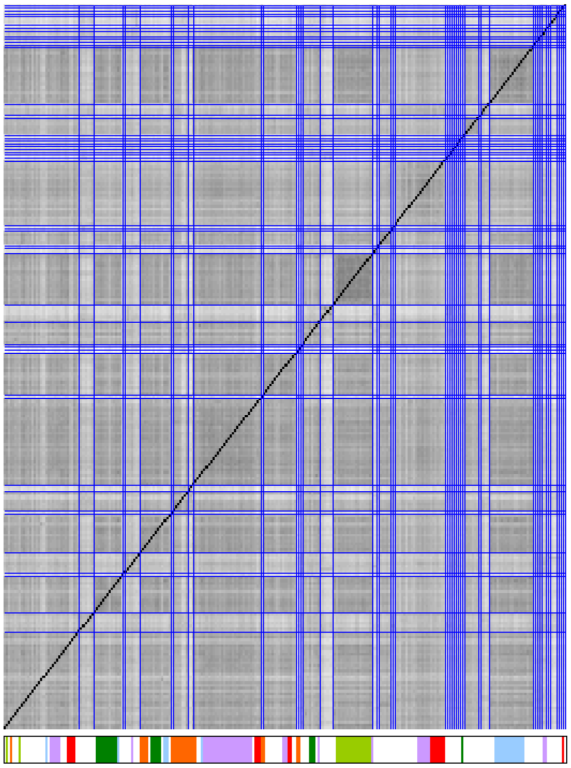

50 Clusters

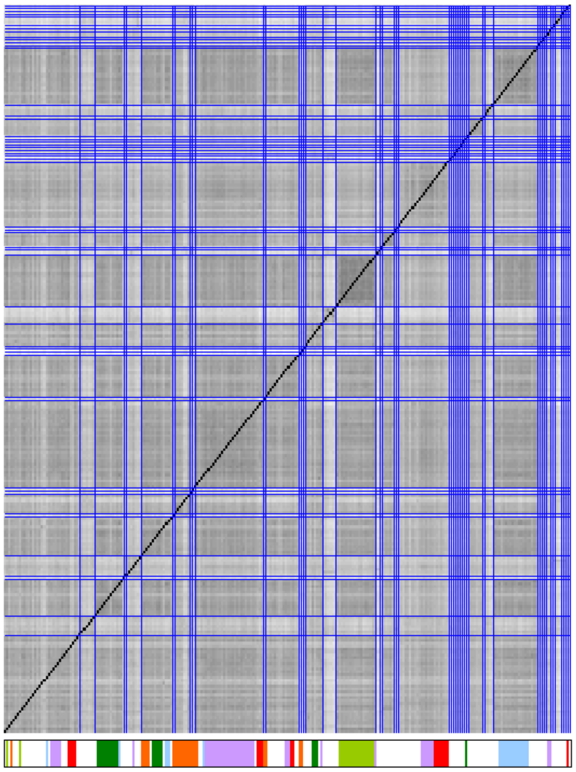

51 Clusters

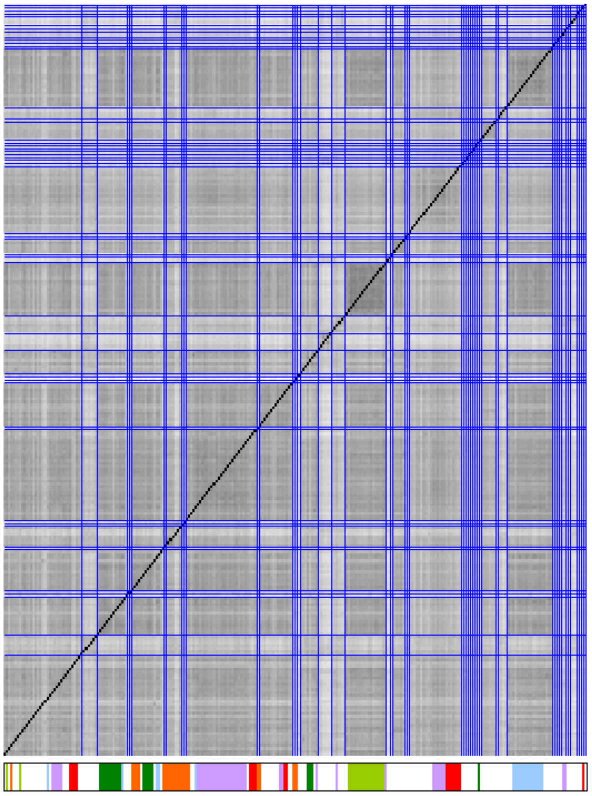

52 Clusters

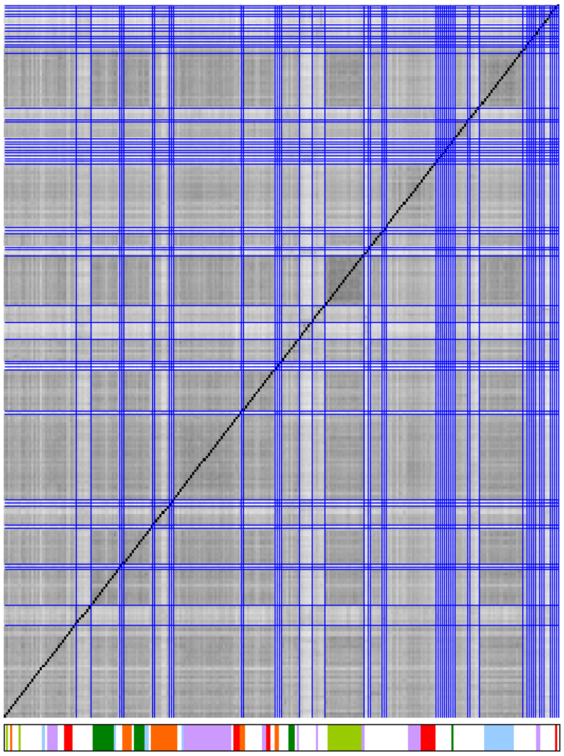

53 Clusters

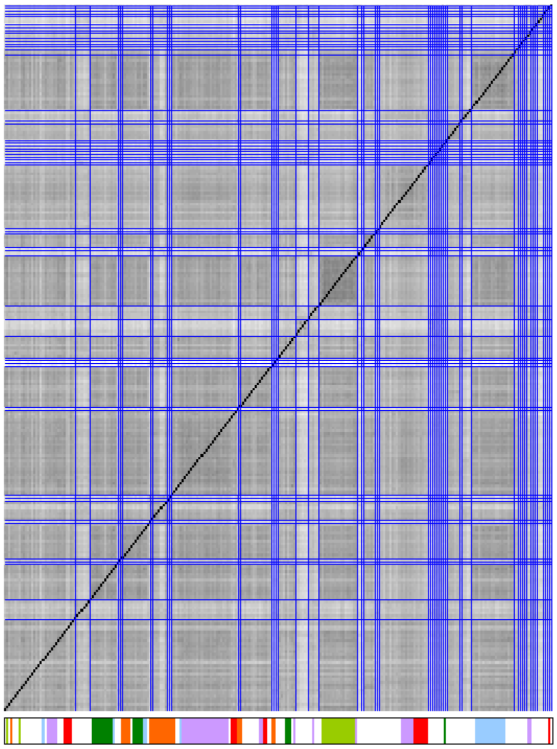

54 Clusters

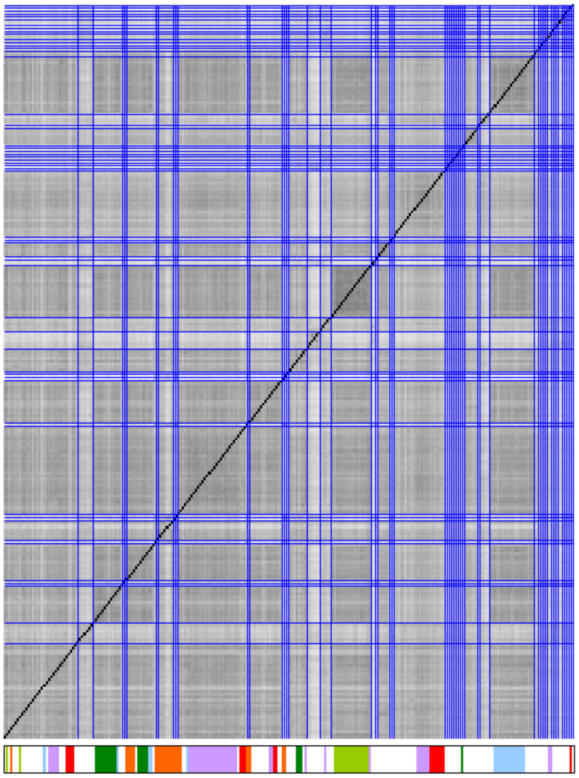

55 Clusters

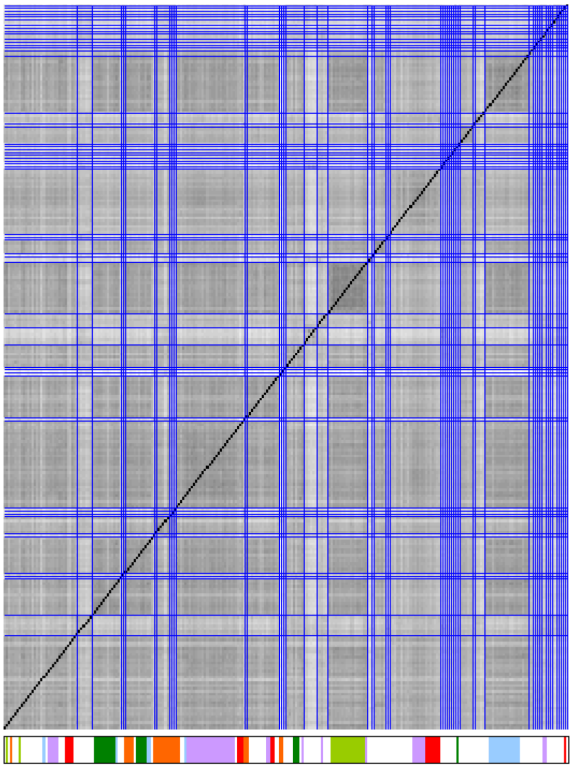

56 Clusters

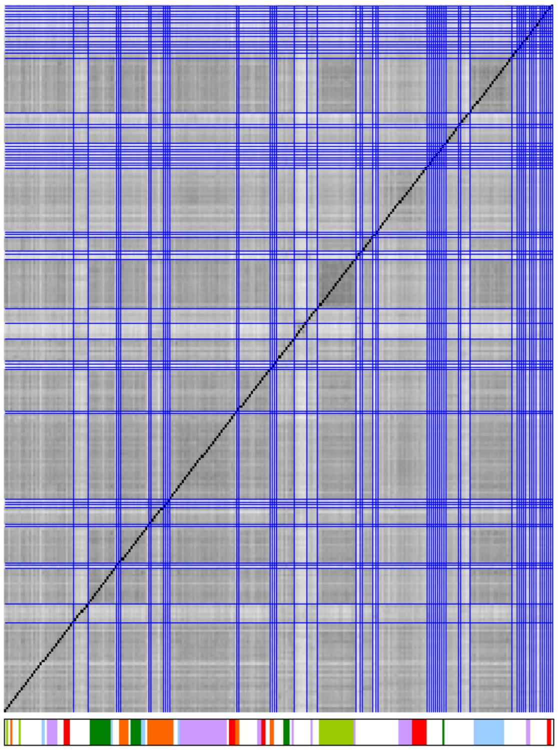

57 Clusters

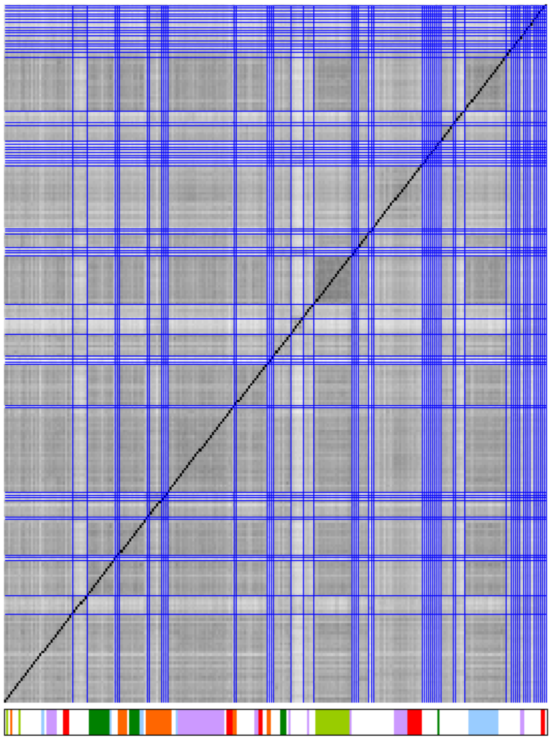

58 Clusters

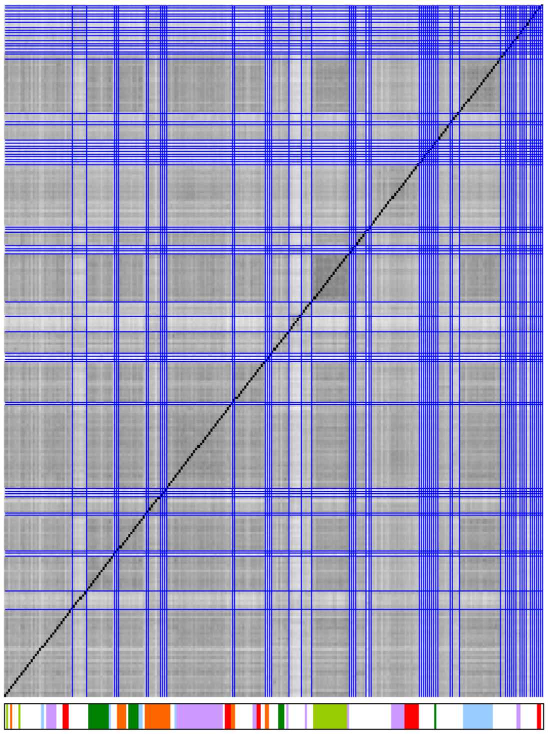

59 Clusters

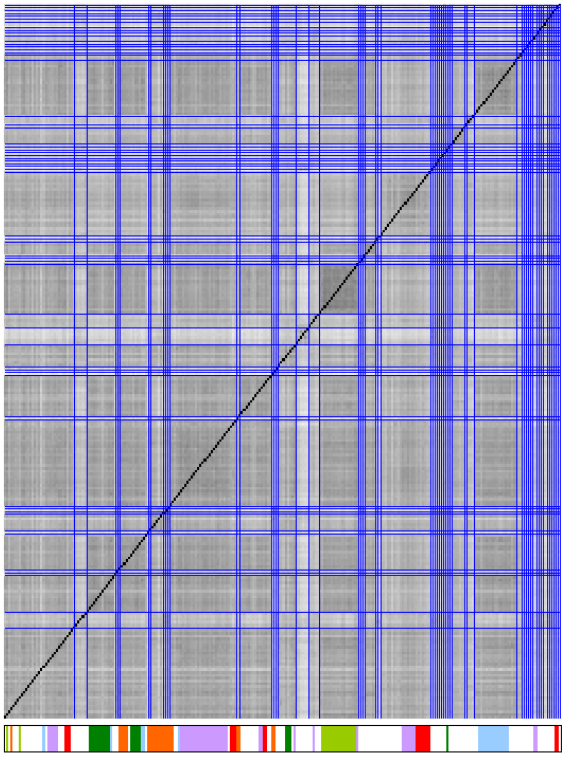

60 Clusters

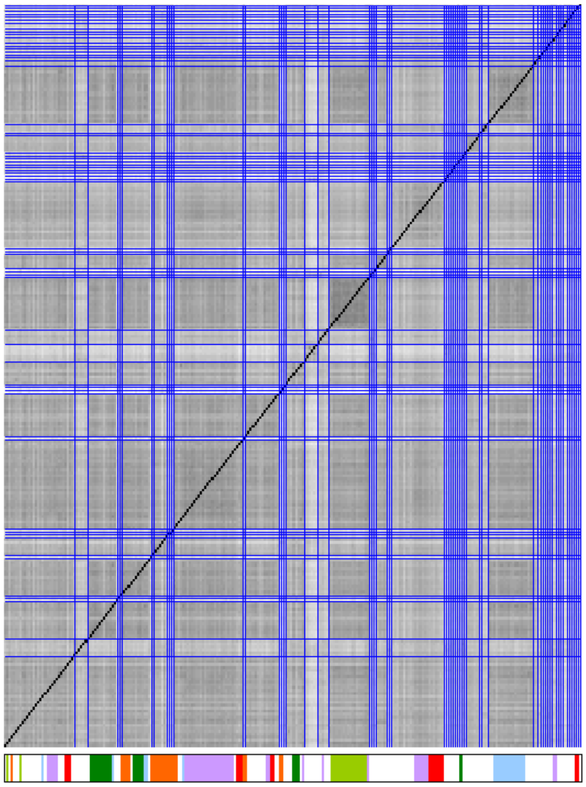

Supplement: File S9 — K-Means clustering of the works using the JSD as a metric. (PDF) [file pone.0111445.s009.pdf]
