# Supplementary material for: An Information Theoretic Clustering Approach for Unveiling Authorship Affinities in Shakespearean Era Plays and Poems
Source: PLoS One. 2014 Oct 27;9(10):e111445. doi: 10.1371/journal.pone.0111445 (PMC4210181; doi:10.1371/journal.pone.0111445)
Supplement: File S8 — Hierarchical clustering of the works using the JSD as a metric. (PDF) [file pone.0111445.s008.pdf]

Cluster Dendrogram, JSD distance

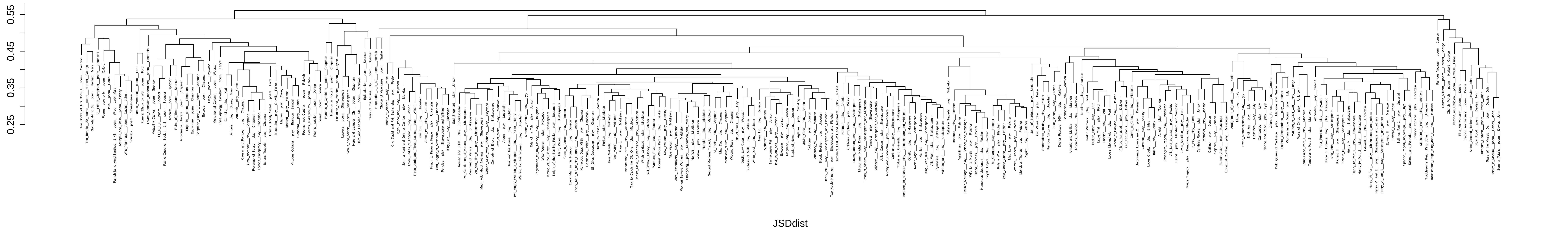

R command used: hclust (JSDdist, "complete")
